# Inner speech in motor cortex and implications for speech neuroprostheses

**DOI:** 10.1016/j.cell.2025.06.015

**Published:** 2025-08-14

**Authors:** Erin M. Kunz, Benyamin Abramovich Krasa, Foram Kamdar, Donald Avansino, Nick Hahn, Seonghyun Yoon, Akansha Singh, Samuel R. Nason-Tomaszewski, Nicholas S. Card, Justin J. Jude, Brandon Jacques, Payton H. Bechefsky, Carrina Iacobacci, Leigh R. Hochberg, Daniel B. Rubin, Ziv M. Williams, David M. Brandman, Sergey D. Stavisky, Nicholas AuYong, Chethan Pandarinath, Shaul Druckmann, Jaimie M. Henderson, Francis R. Willett

**Affiliations:** 1. Department of Electrical Engineering, Stanford University, Stanford, CA, USA; 2. Wu Tsai Neurosciences Institute, Stanford University, Stanford, CA, USA; 3. Neuroscience Graduate Program, Stanford University, Stanford, CA, USA; 4. Department of Neurosurgery, Stanford University, Stanford, CA, USA; 5. Howard Hughes Medical Institute at Stanford University, Stanford, CA, USA; 6. Department of Mathematics, Stanford University, Stanford, CA, USA; 7. Wallace H. Coulter Department of Biomedical Engineering, Emory University and Georgia Institute of Technology, Atlanta, GA, USA; 8. Department of Neurological Surgery, University of California, Davis, CA, USA; 9. School of Engineering and Carney Institute for Brain Sciences, Brown University, Providence, RI, USA; 10. VA Center for Neurorestoration and Neurotechnology, Office of Research and Development, VA Providence, Healthcare System, Providence, RI, USA; 11. Center for Neurotechnology and Neurorecovery, Department of Neurology, Massachusetts General Hospital, Harvard Medical School, Boston, MA, USA; 12. Department of Neurosurgery, Massachusetts General Hospital, Harvard Medical School, Boston, MA, USA; 13. Harvard-MIT Division of Health Sciences and Technology, Boston, MA, USA.; 14. Program in Neuroscience, Harvard Medical School, Boston, MA, USA; 15. Department of Neurosurgery, Emory University, Atlanta, GA, USA; 16. Department of Cell Biology, Emory University, Atlanta, GA, USA; 17. Department of Neurobiology, Stanford University, Stanford, CA, USA

**Keywords:** Inner speech, covert speech, brain-computer interface, speech neuroprosthesis, motor cortex

## Abstract

Speech brain-computer interfaces (BCIs) show promise in restoring communication to people with paralysis^1–3^, but have also prompted discussions regarding their potential to decode private inner speech^4–6^. Separately, inner speech may be a way to bypass the current approach of requiring speech BCI users to physically attempt speech, which is fatiguing and can slow communication. Using multi-unit recordings from four participants, we found that inner speech is robustly represented in motor cortex, and that imagined sentences can be decoded in real-time. The representation of inner speech was highly correlated with attempted speech, though we also identified a neural “motor-intent” dimension that differentiates the two. We investigated the possibility of decoding private inner speech, and found that some aspects of free-form inner speech could be decoded during sequence recall and counting tasks. Finally, we demonstrate high-fidelity strategies that prevent speech BCIs from unintentionally decoding private inner speech.

## In Brief

Inner speech is robustly represented in motor cortex, and can be decoded in real-time to restore communication to people with paralysis. Unintentional decoding of private inner speech can be prevented with high fidelity.

## Graphical Abstract

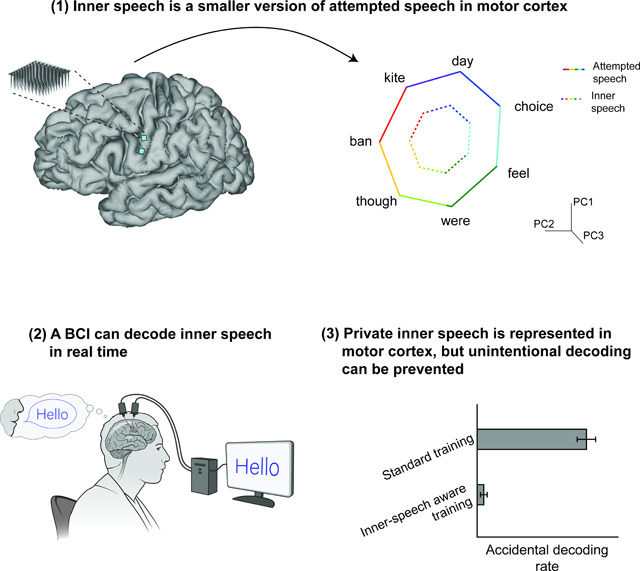

## Introduction

Brain-computer interfaces (BCIs) offer a promising solution for restoring lost movement or communication in people with paralysis due to injury or disease^7^. Successful demonstrations have shown that people with tetraplegia can use neural signals to control a computer cursor^8–13^, manipulate a robotic arm, or even their own arm^14–16^. Recently, BCIs have restored rapid communication through accurate decoding of handwriting^17^ and speech^1,3,18–20^, exceeding the rates offered by alternative devices (e.g., eye tracking). Encouragingly, the most recent demonstration highlights the successful everyday use of a speech neuroprosthesis by a person with ALS for open-ended communication^2^.

Given such rapid progress in speech BCIs, it is important to characterize the limits of what can be decoded from speech motor cortex. One concern by researchers and potential users is the possibility of decoding private inner speech the user doesn’t intend to say aloud. While not everyone associates internal cognition with language, many individuals report experiencing an “inner monologue”^21–24^. Inner speech (also called imagined speech, internal speech, covert speech, silent speech, self-talk, speech imagery, internal monologue or verbal thought) is theorized to support complex cognitive processes including working memory, verbal rehearsal, logical reasoning, executive function, behavioral control and motivation^25–31^. It is also implicated in silent reading, with many people reporting evoked auditory or motor speech imagery while reading^32,33^.

Practical challenges also remain in the translation of speech BCIs. Current systems require users to attempt to produce speech to the best of their ability (“attempted speech”), which can be tiring and may have inherent speed limitations for paralyzed users. A BCI that decodes inner speech, in which users imagine speaking without attempting motor output, could address such issues.

Both neuroimaging and electrophysiological studies have shown that inner speech engages a similar (but not identical) cortical network as physically produced speech^34–36^, raising the possibility that electrodes placed for decoding attempted speech may also enable inner speech decoding^34,37–46^. Precise neural differences between inner and produced speech remain elusive^25,32,47,48^. Neuroprosthetic studies using electrocorticography (ECoG) have shown that inner speech can be decoded from particular regions of the cortex but differ in their conclusions about which regions contribute^45,49–51^. Most recently, Wandelt *et al*. demonstrated inner speech decoding from signals recorded by intracortical microelectrode arrays in supramarginal gyrus (SMG), revealing a shared representation across inner, produced and perceived speech^52^.

Here, we studied the neural representation of inner speech in four BrainGate2 participants with microelectrode arrays placed in motor cortex. We discovered that inner speech is robustly represented, and demonstrated a proof-of-concept real-time inner speech BCI that can decode self-paced imagined sentences from a large vocabulary (125k-words). We also found that aspects of free-form inner speech could be decoded even during tasks where participants were not explicitly instructed to use inner speech. By characterizing its neural geometry, we found that inner speech appears to be a more weakly modulated version of attempted speech, although the two can be distinguished with the help of a neural “motor-intent” dimension. Accordingly, we found that an attempted speech BCI can be trained to ignore inner speech with high accuracy. To prevent unintended output during inner speech BCI use, we also demonstrate a system where an internally spoken “keyword” can be detected with high accuracy, enabling a user to “lock” and “unlock” the system.

## Results

### A spectrum of attempted, inner and perceived speech is represented in motor cortex

To investigate inner speech representation in motor cortex, we analyzed microelectrode recordings from four participants (T12, T15, T16, and T17) during a range of verbal speech behaviors (Table S1). At data collection, T12 and T15 were severely dysarthric due to ALS, T16 was dysarthric from a pontine stroke, and T17 was anarthric and ventilator-dependent due to ALS. T12, T15, and T16 could partially articulate and vocalize, though their speech was unintelligible to untrained listeners, while T17 communicated solely with extraocular muscles.

**Figure 1:**
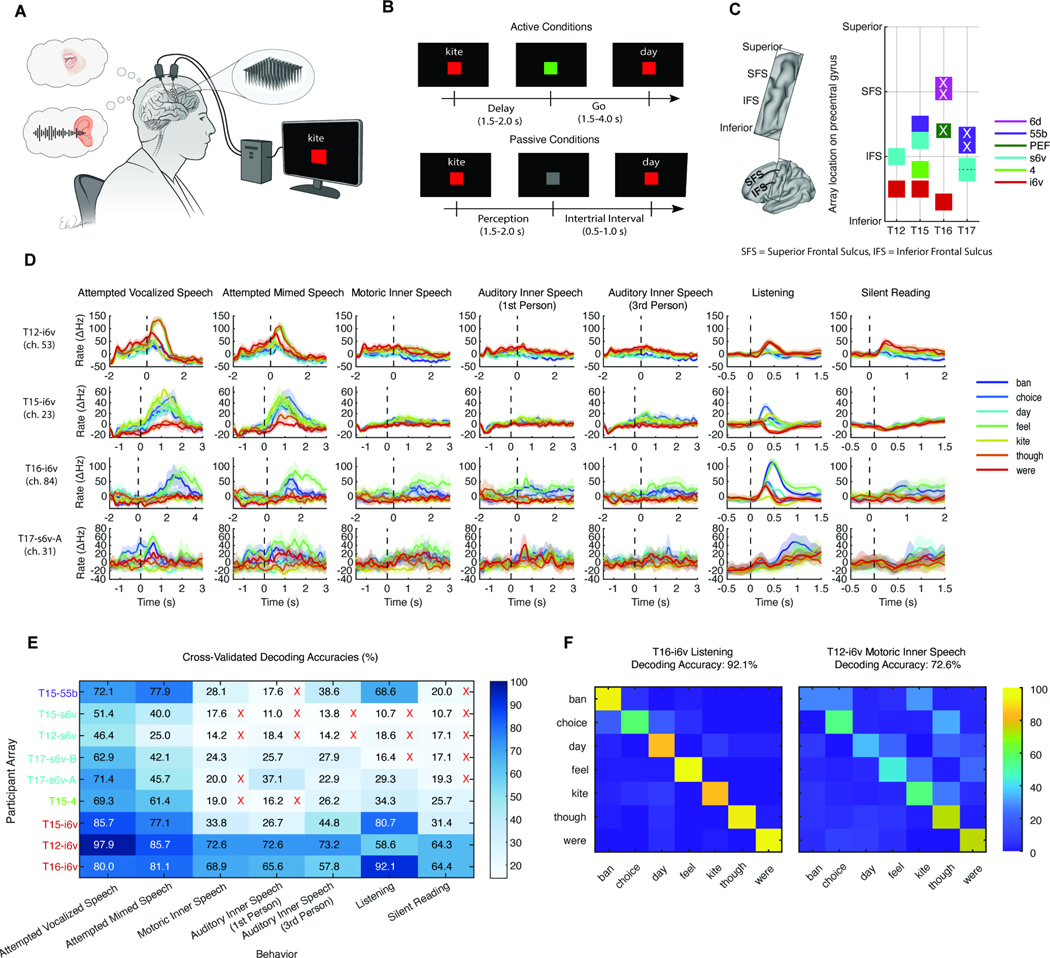
Inner speech, perceived speech and silent reading are represented in ventral and mid precentral gyrus **A)** To assess tuning to different verbal behaviors, neural activity was recorded during attempted speech, inner speech, reading, and listening for a set of 7 words (Table S1,S2). **B)** Example trial structure and visual cues shown on the screen for active (attempted or inner speech) and passive (silent reading or listening) behavior conditions. No text was displayed during listening blocks. **C)** Neural activity was recorded from microelectrode arrays chronically implanted along the precentral gyrus in four participants. A white X indicates that decoding accuracy was not above chance for any behavior (95% confidence interval for accuracy intersected chance) and the array was excluded from further analysis. **D)** The mean firing rate for each cued word for each behavior is shown for an example electrode channel from each participant (estimated from threshold crossings). Shaded regions indicate 95%CIs. **E)** Ten-fold cross-validated decoding accuracy is displayed by array and behavior (Gaussian Naive Bayes, 500ms window); red X’s denote that the 95%CI for accuracy intersected chance level (14.3%). Participant arrays that lacked significance for all behaviors were excluded from further analysis and marked with a white X in C. Notably, while T16’s PEF and 6d arrays recorded spiking activity on many electrodes that were not tuned to speech, T17’s 55b arrays recorded very little spiking activity in general. **F)** Example confusion matrices for T16’s listening trials (92.1% accuracy, 95% CI [86.4%, 96.0%]) and T12’s motoric inner speech trials (72.6% accuracy, 95% CI [65.7%, 78.8%]). See also Figure S1.

We designed task instructions to explore the gradient between attempted (vocalized or mimed) and inner speech, as well as perceived speech and silent reading (Table S1). For inner speech, we assessed three strategies based on reported inner speech experiences^25^. Participants followed visual cues in an instructed-delay task (Figure 1A-B) using a set of seven single-syllable common English words of similar duration and non-overlapping phonemes (see Methods 2.1).

Participants had 2 (T12), 4 (T15, T16) or 6 (T17) microelectrode arrays placed along the precentral gyrus, spanning regions of areas 6v, 4, PEF, 55b, and 6d as defined by individualized cortical parcellations^53^ (Figure 1C). Recordings from each region were analyzed separately to reveal localized differences in neural representation.

Inner speech, perceived speech, and reading were all represented in the precentral gyrus. In three participants, arrays in the inferior area 6v (i6v) decoded word representations above chance (14.3%) across all seven behaviors using a Gaussian Naive Bayes classifier (Figure 1E). T15’s area 4 array (primary motor cortex) weakly represented 3rd Person Auditory Inner Speech, listening, and reading. T17’s superior 6v (s6v) arrays significantly decoded most inner speech conditions—with one array also representing listening—while T15’s 55b array (mid precentral gyrus) significantly decoded two inner speech conditions and listening. Notably, in some participants the decoding accuracy for inner and perceived speech approached or exceeded that for attempted speech. For instance, T16’s listening was decoded at 92.1% (95% CI = [86.4%, 96.0%]) versus 80.0% for attempted vocalized speech, and T12’s motoric inner speech was decoded at 72.6% (95% CI = [65.7%, 78.8%]) versus 97.9% for attempted vocalized speech (Figure 1F). Other sampled regions lacked significant decodability of all speech behaviors and were excluded from further analysis (T16–6d ‘hand knob ’area, T16-PEF premotor eye field, T17–55b mid precentral gyrus). Finally, we found that the neural tuning and decoding performance could not be explained by small differences in word duration (Figure S1), and were likely driven by other features such as differences in phonemic content.

### A shared neural code for attempted, inner, and perceived speech

Having found representations for attempted speech, inner speech, listening and silent reading in the same regions of precentral gyrus, we next investigated the relationship between the neural representations of the same words across behaviors. We considered two hypotheses for how inner speech could be represented within the same neural ensemble as attempted speech while not triggering any motor output. First, attempted and inner speech could lie in orthogonal subspaces^54,55^, allowing for independent encoding of output-potent attempted and output-null inner speech signals, as has been found in motor cortex for arm reaching^56–58^. Alternatively, attempted and inner speech could share the same neural encoding subspace but differ in magnitude, such that inner speech does not reach an activation threshold to generate motor output^59^.

**Figure 2:**
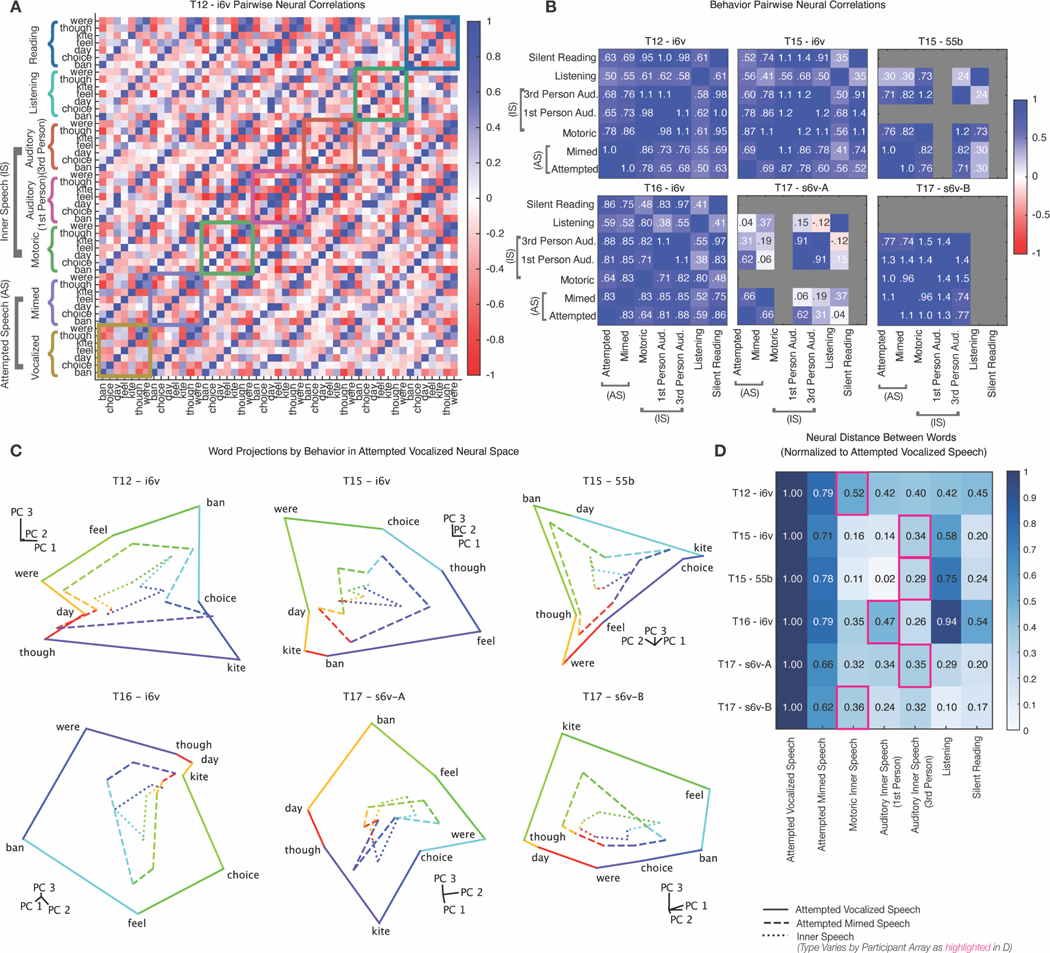
Inner speech and perceived speech as scaled-down versions of attempted speech in motor cortex **A)** Each (i, j) entry in the matrix is the Pearson correlation between the average N × 1 neural feature vectors for word-behavior *i* and *j*, where N is the number of neural features from an array. The off-diagonal banding shows that the same word across behaviors is correlated. Similar across-word correlation patterns also suggest that neural geometry is shared among behaviors; for example, “though” and “were” consistently correlate positively, while “though” and “ban” correlate negatively. A cross-validated metric was used to reduce bias. **B)** Each (*i*, *j*) entry represents the correlation of neural representations across all 7 words for behaviors *i* and *j.* Since a cross-validated estimator of correlation was used, values can be greater than 1 (see Methods). **C)** Projections of average word representations into the subspace defined by the top three principal components for attempted vocalized speech visually demonstrate the shared structure and relative sizes of word representations across attempted and inner speech behaviors. Top 3 PC’s captured 75–82% of variance. **D)** Average neural distances between words within each behavior, normalized to the largest (attempted vocalized speech), represent the modulation magnitude relative to fully attempted speech (e.g., T12-i6v motoric inner speech is about 52% of attempted speech). The pink box highlights the inner speech behavior shown in C.

We measured the overlap between neural representations of attempted and inner speech by correlating window-averaged, neural population firing rate vectors for the same word across different behaviors. Figure 2A displays an example correlation matrix, where strong off-diagonal banding shows that words are encoded similarly across behaviors—including listening and silent reading. We then computed cross-behavior correlations across arrays in regions most responsive to inner speech (Figure 2B). Overall, the correlations were high across behaviors—except in one of T17’s s6v arrays—supporting a shared neural representation of words.

We applied Principal Components Analysis (PCA) to visualize the neural geometry of the seven words in a three-dimensional space (Figure 2C), depicting attempted-vocalized (solid), attempted-mimed (dashed) and inner speech (dotted). The relative positions of words were preserved across behaviors. To quantify the relative size of the representations across behaviors, we measured neural distances between word pairs—larger distances indicating greater separation due to greater modulation—and normalized these by the attempted-vocalized condition, which had the largest modulation. Inner and perceived speech representations were scaled down relative to attempted speech (Figure 2D). This pattern supports the activation threshold hypothesis for preventing motor output during listening, reading, and inner speech.

Notably, T17’s results reflect similar correlations and scale differences as the participants with dysarthria, demonstrating that neural signals in an anarthric, ventilator-dependent person are distinguishable between attempted and inner speech. This finding reveals important insights into the potential efficacy of attempted versus inner speech BCIs for people with anarthria.

### Real-time decoding of self-paced inner speech in three dysarthric individuals

We next investigated whether self-paced inner speech of whole sentences is also represented in motor cortex. To test this, we evaluated the performance of an inner speech BCI that decodes imagined sentences in real-time. Such an approach could be preferred by some BCI users with partial paralysis, since attempted speech requires activation of orofacial muscles and breath control that can be fatiguing and produces distracting vocalizations.

**Figure 3:**
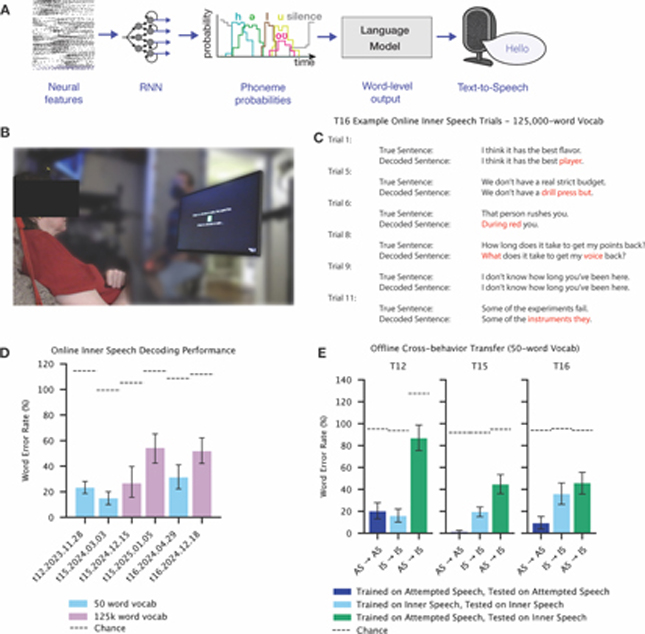
Real-time decoding of self-paced inner speech **A)** Neural features were fed into a recurrent neural network (RNN) that outputs probabilities for 39 phonemes and a silence token every 80 ms. These probabilities were decoded via a language model to yield the most likely word sequence, which was then displayed and converted to audio by a text-to-speech algorithm. **B)** T16 using an inner speech BCI decoding from a large 125k word vocabulary in real-time (Video S1). A text cue appears above the green square and decoded text lies below. **C)** Example decoded sentences from T16’s inner speech from an evaluation block with an overall word error rate of 52% (95% CI: [42.1%, 61.8%]) for a 125,000-word vocabulary. **D)** Word error rates during online inner speech decoding for three participants for either a 50-word (blue) or 125,000-word vocabulary. Chance values are indicated by dashed lines, and denote the lower bound (2.5th percentile) of a chance word error rate distribution calculated by shuffling decoded outputs 100 times with respect to ground truth sentences. Error bars indicate 95% confidence intervals determined via bootstrap resampling (10,000 resamples). **E)** Offline performance of a decoder trained on attempted speech and evaluated on inner speech trials (green bars), compared to baseline decoding performance for attempted speech (dark blue) and inner speech (light blue). Dashed lines show chance levels, and error bars indicate 95% confidence intervals, similarly computed as in D. Note: T12’s outlier cross-decoding error rate is high due to significantly more words being predicted than were cued, including many duplicated words.

Using the decoding pipeline from our previous work^1,2^ (Figure 3A), we trained real-time decoders on data recorded as participants internally spoke cued sentences with their preferred inner speech strategy (see Methods). We combined up to 1 hour of cued inner speech data with previously collected attempted speech data for model training. For an initial feasibility test using a limited 50-word vocabulary, we achieved word error rates (WERs) of 24% (95% CI: [18.5%, 28.0%]), 14% ([9.9%, 20.2%]), and 33% ([21.8%, 41.1%]) for T12, T15, and T16, respectively (Figure 3D, light blue). We also evaluated real-time inner speech decoding using a large 125,000-word vocabulary with T15 and T16 (sentences from the Switchboard corpus), achieving WERs from 26% ([15.5%, 32.3%]) to 54% ([42.5%, 65.3%]) (Figure 3D, pink, Video S1). This demonstrates the potential of an online, self-paced inner speech neuroprosthesis in severely dysarthric participants using a large vocabulary. All participants had experience with attempted speech decoding and preferred inner speech for its lower physical effort and more neutral outward appearance.

Finally, to probe the relationship between attempted and inner speech and assess whether inner speech could be decoded by an attempted-speech BCI, we collected the same training sentence set from the 50-word vocabulary under both conditions (attempted and inner speech). Offline decoders trained on these datasets showed that attempted-speech decoding was generally better (T15 and T16; non-intersecting 95% CIs) or on par (T12; intersecting 95% CIs) with inner speech.

Notably, when evaluating inner speech with a decoder trained solely on attempted speech, performance was above chance for all participants (Figure 3E, green), indicating that a speech BCI trained only on attempted speech signals can decode inner speech.

### Uninstructed inner speech during a sequence recall task can be decoded from motor cortex

**Figure 4:**
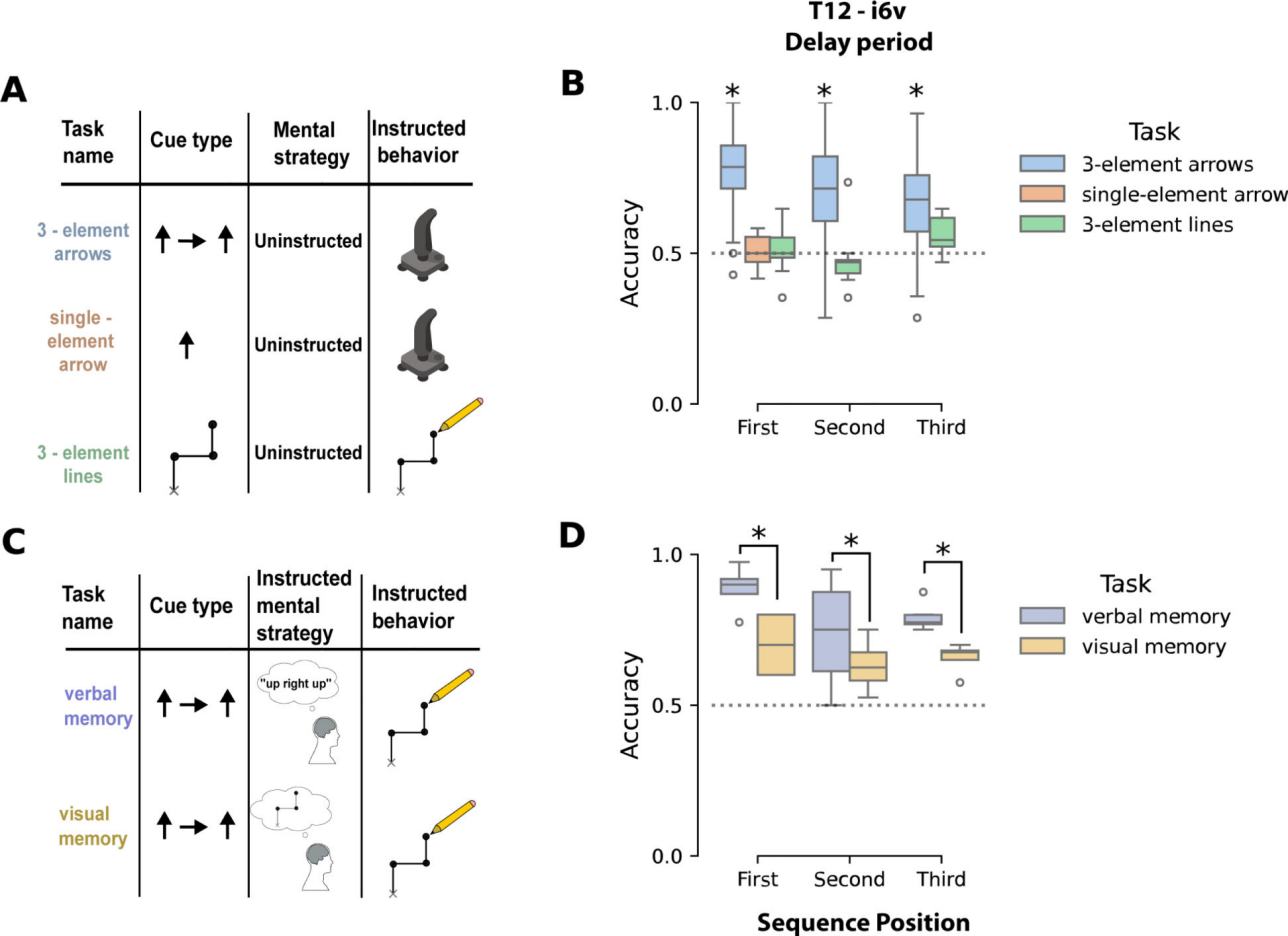
Uninstructed inner speech elicited by a serial recall task can be decoded from i6v. **A)** T12 performed three upper extremity motor tasks with varied cues and memory demands to elicit verbal short-term memory without explicit mental strategy instructions. The 3-element arrows task was designed to prompt verbal memory (eliciting inner speech) for serial recall, while the single-element arrow and 3-element lines tasks served as controls (designed not to elicit inner speech). **B)** Sequence position decodability was measured by training binary Linear Discriminant Analysis models to classify sequence pairs that differed in one position (e.g., first position: ↑ → ↑ vs ↓ → ↑) using i6v neural activity from a 2-second delay period (pre-go) window. Box plots show cross-validated accuracy (dotted line indicates chance). Only the 3-element arrows task produced significant decoding in all three positions (bootstrap-derived CIs vs chance level of 0.5). **C)** Two versions of the serial recall task with explicit instructions to use either a verbal or visual short-term memory strategy (mental strategy instructions refer to how to memorize the sequence, while instructed behavior refers to the motor output during recall). **D)** Same as B but for tasks that only differed in instructed mental strategy. Decoding accuracy significantly increased in all sequence positions when T12 engaged in a verbal memory strategy. Significance assessed via bootstrap-derived confidence intervals of increase in decoding accuracy due to verbal memory instruction, compared to chance level of zero (i.e. no difference between verbal and visual memory). See also Figures S2, S3

Thus far, we have studied explicitly cued inner speech, but its representation could be distinct from that of uninstructed, private inner speech. To investigate this, we conducted a series of sequential recall tasks with T12, hypothesizing they would naturally elicit inner speech without explicit instruction. Prior research suggests that cue type influences inner speech use^60^ and that verbal short-term memory is commonly used to retain sequential information^61^. Therefore, we designed three upper extremity tasks with varied visual cues to differentially engage inner speech, without providing any explicit instructions regarding mental strategy (Figure 4A).

In the first task, T12 memorized a sequence of three arrows during a delay and then moved a joystick in those directions (Figure 4A, “3-element arrows”). We hypothesized that the symbolic arrow cues and three-element sequence could trigger inner speech as a mnemonic (e.g. mentally repeating “up right up” for ↑ → ↑). Binary decoders were trained to distinguish cues that differed in one position (e.g., above-chance performance for ↑ → ↑ versus ↓ → ↑ would indicate representation of the first element). In area i6v, all three sequence positions were decoded above chance—likely due to T12’s use of inner speech (Figure 4B; 95% CI did not include chance level of 0.5). To rule out the possibility that these results were due to hand-motor planning, we tested two additional tasks designed not to elicit inner speech.

First, we tested a single-element arrow task which, due to its simplicity, we predicted would not engage inner speech (Figure 4A). Decoding analysis in T12-i6v revealed that the cued direction was not decodable above chance—despite identical hand-motor movements (Figure 4B, CI did not cross 0.5)—indicating that the i6v tuning in the 3-element sequence was not merely due to motor preparation or symbolic cues. Next, we designed a third task (3-element lines) to evoke sequential hand movements without inner speech. Here, an ordered sequence of line segments was presented as an image, which T12 attempted to reproduce. We hypothesized that this geometric cue would rely on visual short-term memory rather than inner speech. Consistent with this, movement directions were not decoded above chance (Figure 4B, CI overlapped with chance). Together, these tasks suggest that the neural representation observed in the 3-element arrows task likely reflects uninstructed inner speech rather than general hand-motor planning.

To further test our hypothesis that the 3-element arrow cues naturally elicited inner speech, T12 performed the task while explicitly instructed to use either a verbal or visual memory strategy (Figure 4C). T12 achieved 100% recall accuracy and reported confidence in switching between strategies. In i6v, tuning increased significantly at all sequence positions when using the verbal strategy (Figure 4D, CI did not cross 0). Finally, we found that decoders fit directly to attempted speech of direction words performed above chance when assessed on delay period activity when T12 used a verbal strategy (Figure S2), further indicating that the effects we observed were due to inner speech.

Replication with T16 yielded similar, though weaker, effects that occurred only during the go period (Figure S3). This suggests individual variation in the encoding strength and timing of verbal short-term memory in i6v.

### Aspects of uninstructed inner speech can be decoded during counting and prompted thinking tasks

**Figure 5:**
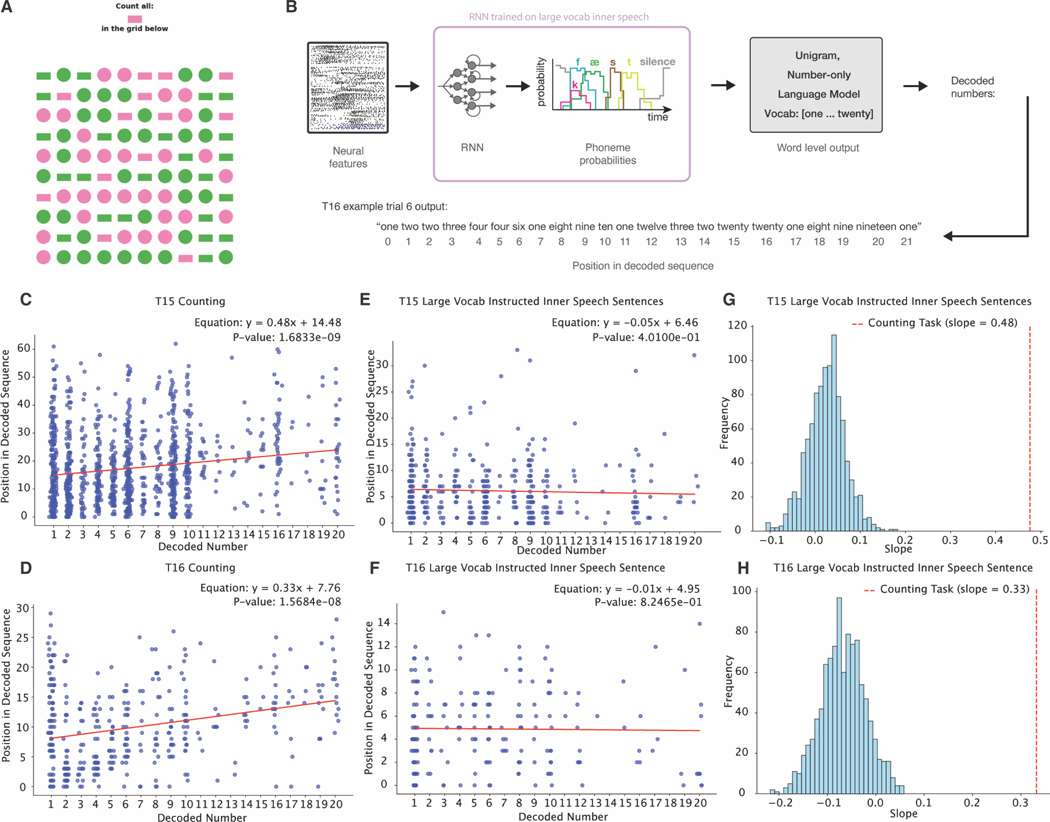
Neural activity recorded during a counting task can be decoded into a sequence of increasing numbers **A)** Neural activity was recorded while participants performed a conjunctive counting task. Participants were instructed to silently count a specified shape and color, and then speak the number aloud during a separate "go" epoch. No specific mental strategy was instructed. **B)** The neural activity during the "counting" epoch was passed through an inner speech RNN decoder, which was trained on a 125,000-word vocabulary from the same session. Instead of using a standard language model, a unigram language model trained only on number words (one to twenty) was used to generate word-level outputs. This model could only produce number words between one and twenty, and word output was independent of surrounding context, unlike larger models that use language statistics to predict words based on prior and subsequent words. **C,D)** For T15 and T16, decoded numbers showed a significant positive correlation with their position in the sequence (T15: slope = 0.48, p = 1.69×10⁻⁹; T16: slope = 0.33, p = 4.84×10⁻⁹), indicating sequential increases. Jitter was added along the x-axis for visualization. **E,F)** Same as C,D except neural activity was recorded during instructed inner speaking of sentences from the Switchboard corpus (collected as training data for the RNN). Lack of significant slopes provides evidence that increasing sequences of numbers in C,D are not likely to be decoded by chance. **G)** Histogram of slopes obtained from regression analyses of 1,000 resamples of T15’s large-vocabulary Switchboard inner speech sentences, with a red dashed line indicating the slope for the counting task. This shows that numbers decoded from the counting task sequentially increase significantly more than when the same analysis is performed on instructed inner speech trials using the Switchboard corpus **H)** Same as G, but for T16. See also Figures S4, S5

To further probe whether uninstructed inner speech could be decoded, we designed a conjunctive-counting task with no explicit instruction on whether or how to engage inner speech. Participants viewed a grid of shapes in two colors and were asked to count instances of a specific colored shape (Figure 5A). We hypothesized that visual distractors would lead participants to scan the grid and engage in sequential inner speech counting^62^. An RNN decoder—trained on instructed inner speech—predicted phoneme sequences during counting, and a unigram, number-only language model was used to generate word sequences (Figure 5B).

Due to being unigram, each number was predicted independently of its neighboring context, such that the predicted word sequence was not biased towards predicting sequences of increasing numbers. For both T15 and T16, decoded numbers increased over the course of the counting trials (Figure 5C: slope=0.48, p-value = 1.69 × 10^−09^; D: slope=0.33, p-value = 4.84 × 10^−09^), supporting the hypothesis that participants engaged in inner speech counting. As a control, the same analysis was performed on trials of instructed inner speech sentences to estimate a null distribution. In this context, no relationship was found between decoded numbers and word position (Figure 5E-H). Additionally, when using the same 5-gram large-vocabulary language model used for closed-loop attempted speech decoding (as opposed to the unigram model), significantly more numbers were decoded during counting task trials (T15: 0.45, 95% CI = [0.2, 0.75]; T16: 2.0, 95% CI = [0.85,3.45]) as opposed to the instructed inner speech Switchboard sentences task (T15: 0.0, 95% CI = [0.0,0.0]; T16: 0.09, 95% CI = [0.0,0.2]) (Figure S4). These results further demonstrate that aspects of private inner speech can be decoded by a speech BCI during free-form tasks in which the user naturally engages in inner speech.

To further investigate scenarios in which decoding private inner speech may be possible, participants were prompted to reflect on verbal or autobiographical details (e.g., “think about your favorite quote from a movie” versus “think about your favorite food”) or to “clear your mind”. Neural activity during thinking was decoded offline using the real-time inner speech decoder from the same day. Significantly more words were decoded during verbal thought prompt responses than when prompted to “clear your mind” (Figure S5). Although most decoded sentences were largely gibberish with occasional plausible phrases, we refrain from reporting the specific outputs given the uncertainty about their representativeness of the participants ’actual thoughts.

### Evidence of a robust motor-intent dimension separating attempted and inner speech

The shared neural code for inner and attempted speech raises the possibility that an attempted-speech BCI could unintentionally decode private inner speech. However, in our results above that showed high neural correlations between attempted and inner speech behaviors (Figures 2), behaviors were recorded in sequential experimental “blocks.” This means that any differences in average firing rates observed between two behaviors collected in separate blocks could be due to spurious drifts in firing rates known to occur across time^63,64^. Because of this, we could not conclude if there were large differences in mean firing rate across behaviors that could help a decoder to distinguish between attempted and inner speech. To test for this, we conducted a follow-up experiment in which both attempted and inner speech trials of the seven words were randomly interleaved within the same experimental blocks, accounting for any nonstationarity, and making it possible to conclusively test for differences in mean firing rates across behaviors.

We first visualized the relative structure of all 14 interleaved conditions (7 words for each behavior) using the top three PCA components (capturing 62%–86% of the variance). The low-dimensional projections confirmed that inner speech shares the same relative word structure as attempted speech, albeit at a reduced scale. Rotating the projection also revealed strong separability between behaviors along a “motor-intent” dimension that describes differences in average (“baseline”) neural activity between the behaviors. Notably, T17’s data aligned with the other participants, suggesting that motor-intention encoding remains intact even in anarthric individuals, for whom there are no differences in observable behavior output between attempted and inner speech.

**Figure 6:**
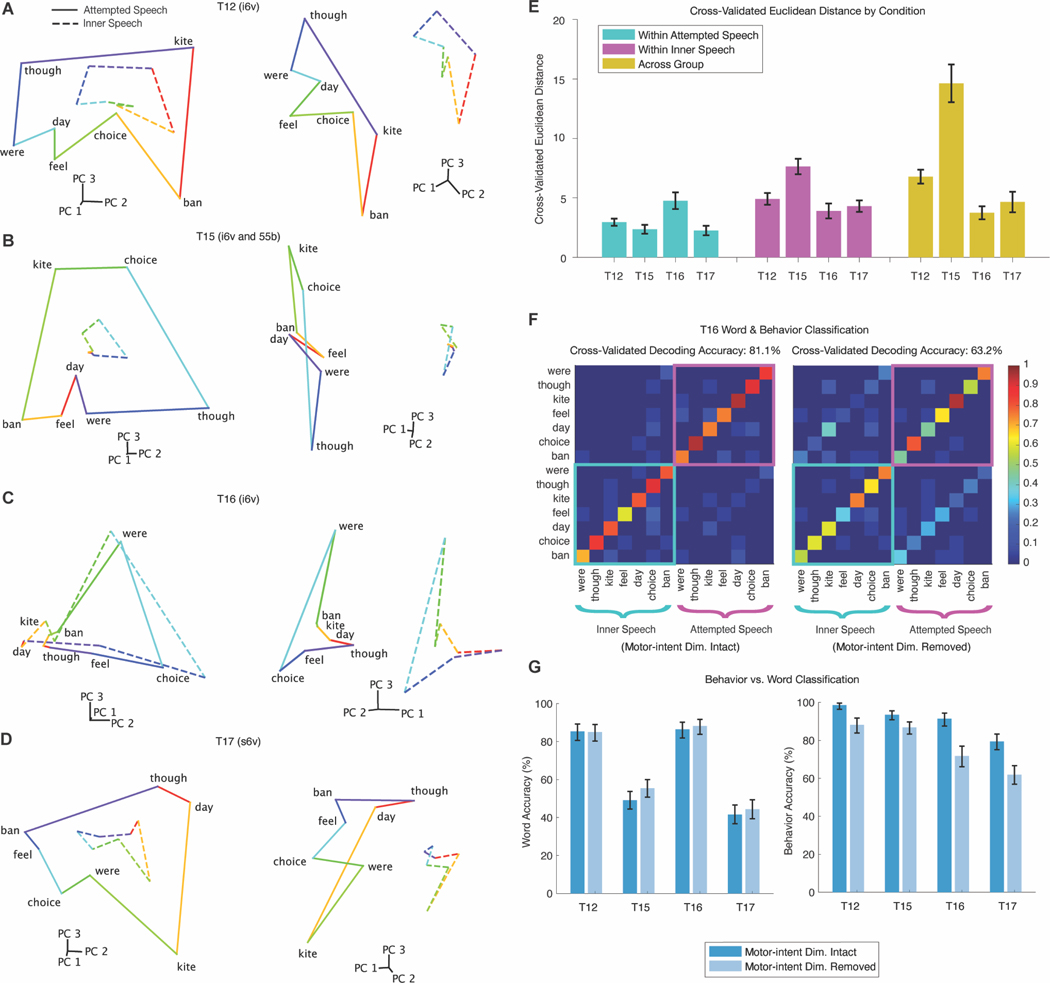
Motor cortex contains a neural dimension representing motor intention that can help distinguish attempted speech from inner speech **A**) For T12, PCA projections of all 14 conditions (7 words each for attempted and inner speech) show that the concentric view (left) reveals shared word structure (attempted: solid; inner: dashed), while the rotated view (right) highlights a clear separation along the motor-intent dimension—defined as the vector between each behavior’s centroids (see Methods 8.4). **B**-**D**) PCA projections for T15,T16, and T17. **E)** Cross-validated Euclidean distances reveal that word-related modulation (within behaviors, turquoise/pink) is similar to (T12, T16, T17) or smaller than (T15) the motor-intent modulation (across behaviors, yellow). **F)** Example confusion matrices for T16 show that removing the motor-intent dimension increases cross-behavior confusion. **G)** Left: Word accuracy (indicating correct word decoding irrespective of the decoded behavior) remains similar before and after removal of the motor-intent dimension (95% confidence intervals intersect). Right: Behavior accuracy (indicating correct behavior decoding irrespective of the decoded word) drops significantly after removal (non-overlapping 95% confidence intervals for all participants).

To quantify the effect of the motor-intent dimension, we compared the Euclidean distances between word pairs within each behavior (word-related modulation) to those between matching word pairs across behaviors (motor-intent modulation, Figure 6E). Motor-intent modulation was comparable to (T12, T16, T17) or greater than (T15) word-related modulation, indicating that it is a robust feature of the neural activity that could help decoders to distinguish between attempted and inner speech.

Next, we trained Gaussian Naive Bayes decoders to distinguish all 14 conditions, once with the motor-intent dimension intact and once with it removed (by projecting it out of the neural features). Here, we define the “motor-intent” dimension as the direction of a vector connecting the centroids of the word representations for each behavior (Methods 8.4). With the motor-intent signal intact, decoders showed little confusion between behaviors. Removing it increased confusion for matching words across behaviors (Figure 6F), although overall decoding performance remained relatively high—potentially due to remaining differences in the sizes of the word representations. Finally, we confirmed that removing the motor-intent dimension left word accuracy largely unchanged while significantly reducing behavior accuracy (Figure 6G), demonstrating that the removed signal carried predominantly behavior-specific information.

### Unintentional decoding of private inner speech can be prevented with high accuracy

**Figure 7:**
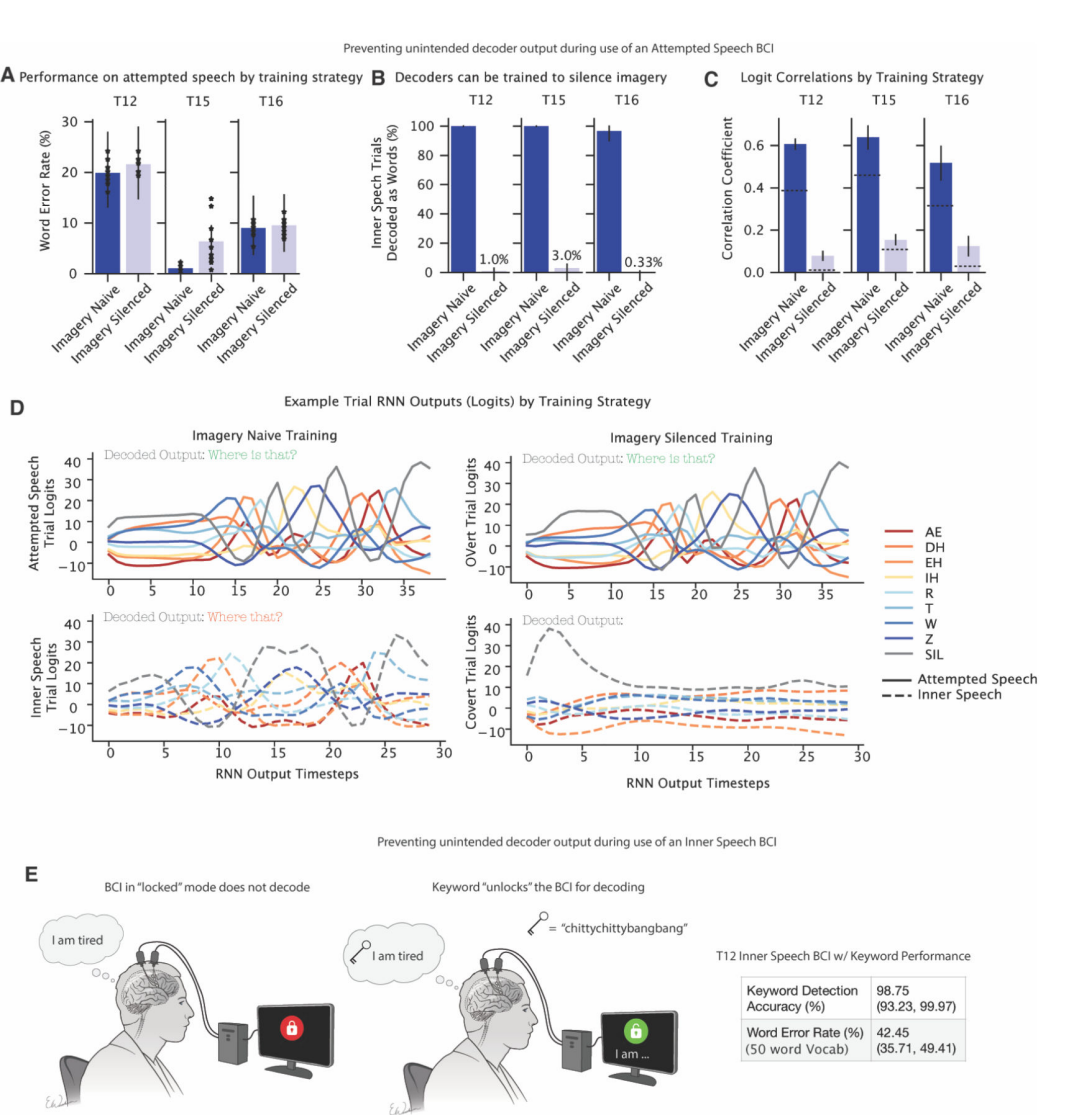
Simple strategies can robustly prevent private inner speech from being decoded by a speech BCI. **A**) The imagery-silenced strategy augments the standard imagery-naive approach (which uses only attempted speech trials) by including inner speech trials labeled as silence. This strategy largely preserves offline decoding performance (measured by word error rate) on attempted speech trials, as indicated by dots (10 RNN training seeds) with 95% confidence intervals. **B**) Imagery-silenced training robustly prevents false outputs during inner speech. **C**) Correlations between RNN outputs for matched inner and attempted speech sentences are much higher with imagery-naive training than with imagery-silenced training (dotted lines show chance-level correlations). **D)** Visualizations of phoneme logit outputs for a T16 sentence illustrate that, in the imagery-naive strategy (left), attempted (top) and inner speech (bottom) produce similar outputs, while the imagery-silenced strategy (right) correctly quiets the output on inner speech trials. **E)** With the keyword strategy, the inner-speech BCI remains in a "locked" mode and does not decode until the unlocking keyword "chittychittybangbang" is detected. In real-time tests with T12, this approach achieved a keyword detection accuracy of 98.75% and a word error rate of 43.45% (95% CI: [35.7%, 49.4%]) for a 50-word vocabulary.

Motivated by the results above, we tested strategies to prevent speech BCIs from inadvertently decoding private inner speech. First, we developed an “imagery-silenced” decoder training strategy. In this approach, an RNN was trained on attempted speech sentences labeled with their phonemes and inner speech sentences labeled as a “silence” token. This differs from current state-of-the-art BCIs that use only attempted speech (“imagery-naive” training). Offline testing showed that the imagery-silenced strategy maintained decoding performance for attempted speech (Figure 7A) while effectively preventing decoding during inner speech trials (Figure 7B). Analysis of the logit outputs revealed that, in the imagery-naive model, logits for matching attempted and inner speech sentences were highly correlated, whereas they were much less correlated in the imagery-silenced model (Figure 7C). Individual trial analyses further demonstrated that the imagery-silenced strategy dramatically quiets RNN output on inner speech trials (Figure 7D), although this strategy remains to be verified in online, real-time decoding.

Next, we tested a keyword strategy to prevent unintended outputs when using an inner-speech BCI. In this strategy, the system only decodes a volitional inner speech utterance if it is first “unlocked” by detecting an inner speech keyword (Figure 7E). In real-time tests with T12, the keyword was correctly detected (or correctly not detected) with 98.75% accuracy (95% CI: 93.23%-99.97%). Together, these results suggest that for both attempted and inner speech BCI, high-fidelity methods exist to prevent unintended decoding of private inner speech.

## Discussion

### Inner speech representations in motor cortex

Speech is a complex behavior engaging multiple cortical regions in a "speech network." Differing from traditional views that posit separate speech perception and speech production areas ^65–67^, recent studies have shown that the neural mechanisms of speech production and perception are connected and overlapping, even in motor areas^68–71^. Studies on inner speech have also shown overlapping mechanisms between attempted and inner speech production ^34,45,49–52^. However, these studies disagree on the degree of overlap and role of the speech motor cortex.

With the level of spatial resolution achieved with microelectrode array recording in four participants, this work demonstrates that there are localized regions of the motor cortex - the middle (55b) and most ventral (i6v) regions of precentral gyrus - for which inner speech, silent reading, and passive listening are robustly represented and appear to be correlated, scaled-down versions of attempted speech. This aligns with previous speech BCI studies in which the middle precentral gyrus^3^ and most ventral precentral gyrus^1,2^ demonstrated the greatest signal contributions to speech decoding.

These “speech hotspot” regions (areas 55b and i6v) strongly represent a spectrum of verbal behaviors, prompting the question: how is motor output inhibited during inner speech? One hypothesis is that attempted and inner speech occupy orthogonal neural subspaces^54,55^, allowing independent encoding of output-potent attempted and output-null inner speech signals—as seen in reaching tasks^56–58^. However, our findings show that the neural representations of attempted, inner speech, and even listening are highly correlated and exist in a shared neural space. These shared signals could represent an abstract sensorimotor or auditory “goal” or target signal used by downstream motor areas, where output gating could occur instead. Alternatively, the weaker modulation during inner speech might simply fail to reach the activation threshold for motor output^59^, or unique aspects of attempted behavior (such as the “motor-intent” dimension we identified) might be used to gate output, as recently proposed for imagined wrist movement in hand motor cortex^72^.

### Implications for Intracortical Speech BCIs

Recent progress in speech neuroprostheses has prompted discussions regarding the extent to which private inner speech may be accessible via neural recordings from speech-related areas of motor cortex. Indeed, a concern raised by both researchers and potential users is “mental privacy” - specifically whether a speech BCI would “be able to read into thoughts or internal monologues of users when attempting to decode (motor) speech intentions”^6^. We show that careful decoder training for attempted speech BCIs, with the help of the “motor intent” dimension, can prevent leakage of inner speech with high fidelity (although high specificity has also been demonstrated without such design^2^).

Inner speech could also be a way to improve speech BCIs, which have so far relied only on attempted speech. In this work, we demonstrated a real-time inner-speech BCI in three people with severe dysarthria. Compared to attempted speech, the inner-speech BCI required less effort, offered improved comfort, and bypassed physiological constraints (e.g., breathing control) that slow attempted speech in people with paralysis, potentially enabling a path forward for speech BCIs to achieve speeds comparable to normal speech. However, inner-speech BCIs may require additional design considerations to prevent accidental “leakage” of inner thought into BCI output; we addressed this by using a simple keyword mechanism for the user to unlock decoding only when intended, achieving high accuracy.

Notably, we also assessed attempted and inner speech in an anarthric person with only extraocular movements and no speech articulator control (T17). Although both conditions produced no observable movement, attempted speech was still more strongly represented, indicating that the volition to articulate is maintained in the motor cortex even in anarthria. This suggests attempted speech may remain a useful behavior to drive speech BCIs in nearly or completely locked-in individuals.

Finally, we demonstrated that aspects of inner speech are decodable even during tasks where it was not explicitly instructed (sequence recall, counting, and prompted thought). Inner speech as a cognitive tool in adults has been implicated in task switching, planning, propositional reasoning, reasoning about others, spatial orientation, categorization, cognitive control, and reading^25^. The extent to which inner speech can be decoded from motor cortex broadly in these varying contexts remains unknown and requires further study. Furthermore, the degree to which speech and language are used for thought is under debate^73,74^. The scope for potential decoding may be limited to concrete mental strategies such as verbal memory, counting, or explicitly verbal thought (e.g., recalling lyrics to a song). Private inner monologue likely differs between individuals and may not unravel concretely, which could make it difficult or impossible to decode from motor cortex.

### Limitations of the study

While we demonstrated that neural activity in motor cortex encodes attempted and various inner speech behaviors similarly in 4 participants, it is unclear whether these findings generalize to others due to the limited sample size and potential variability in individuals ’engagement of inner speech for cognitive tasks. The strategies shown here to ensure mental privacy for individuals using a speech BCI are initial explorations. Additional measures may be needed as speech BCIs become more widely used. Finally, we have shown that instructed inner speech can be decoded using a large vocabulary with word error rates between 26–54%, and that some aspects of free-form inner speech can be decoded in sequence recall, counting, and prompted thinking tasks. However, it was not possible to accurately decode complete, intelligible sentences during free-form thinking. While it may be possible to do so as recording technology improves, it has not yet been demonstrated.

## Resource availability

### Lead contact

Requests for further information and resources should be directed to and will be fulfilled by the lead contact, Erin Kunz (ekunz@stanford.edu)

### Materials Availability

This study did not generate new reagents.

### Data and Code Availability

- Neural data needed to reproduce the findings in this study are publicly available on Dryad [DOI] as of the date of publication.
- All original code has been deposited at [repo] at [DOI] at and is publicly available as of the date of publication.
- Any additional information required to reanalyze the data reported in this paper is available from the lead contact upon request.

## STAR METHODS

### EXPERIMENTAL MODEL AND STUDY PARTICIPANT DETAILS

Data from four participants, referred to as T12, T15, T16, and T17, are reported in this study, all of whom gave informed consent and were enrolled in the BrainGate2 Neural Interface System pilot clinical trial (ClinicalTrials.gov Identifier: NCT00912041, registered June 3, 2009). Approval for this pilot clinical trial was granted under an Investigational Device Exemption (IDE) by the US Food and Drug Administration (Investigational Device Exemption #G090003), as well as the Institutional Review Boards of Stanford University (protocol #52060), University of California Davis, Emory University (protocol #STUDY00003070), and VA Providence Healthcare System (IRB-2011–009). T16 gave consent to publish photographs and videos containing her likeness. All relevant guidelines and regulations were strictly upheld.

T12, a left-handed woman, was 68 years old at the time of data collection, with slowly-progressive bulbar-onset Amyotrophic Lateral Sclerosis (ALS) diagnosed at age 59 (ALS-FRS score of 26 at the time of study enrollment). In March 2022, four 64-channel, 1.5 mm-length silicon micro electrode arrays coated with sputtered iridium oxide (Blackrock Microsystems, Salt lake City, UT) were implanted in T12’s left hemisphere, based on preoperative anatomical and functional magnetic resonance imaging (MRI) and individualized Human Connectome Project (HCP) cortical parcellation (see ^1^ for details). Two arrays were placed in HCP-identified area 6v (orofacial motor cortex) of ventral precentral gyrus, and two were placed in HCP-identified area 44 of inferior frontal gyrus (considered part of Broca’s area). Data are reported from post-implant days 412–995. At the time of data collection, T12 was severely dysarthric for nearly 8 years due to bulbar ALS. She retained partial use of her limbs, and communicated primarily through use of a writing board or iPad tablet. She was able to vocalize while attempting to speak, and was able to produce some subjectively differentiable vowel sounds. However, we had difficulty discerning nearly all consonants produced in isolation and could not reliably make out any consonants or vowels when T12 attempted to speak full sentences at a fluent rate.

T15, a left-handed man, was 45 years old at the time of data collection, with ALS diagnosed at the age of 40. In July 2023, four 64-channel, 1.5 mm-length silicon microelectrode arrays coated with sputtered iridium oxide (Blackrock Microsystems, Salt lake City, UT) were implanted in T15’s left hemisphere, based on preoperative anatomical and functional magnetic resonance imaging (MRI) and HCP individualized cortical parcellation (see ^2^ for details). Two arrays were placed in HCP-identified area 6v (orofacial motor cortex) of ventral precentral gyrus, one in HCP-identified area 55b, and one in HCP-identified primary motor cortex (area 4). Data are reported from post-implant days 230–538. T15 had no functional use of his upper and lower extremities and had severe dysarthria (ALS-FRS score of 23 at the time of study enrollment).

T16, a right-handed woman, was 52 years of age at the time of this study, with tetraplegia and dysarthria due to a pontine stroke approximately 19 years prior to enrollment in the BrainGate2 pilot clinical trial. In December 2023, T16 had four 64-channel intracortical microelectrode arrays (Blackrock Microsystems, Salt Lake City, UT; 1.5 mm electrode length) placed in her left precentral gyrus, guided by individualized HCP cortical parcellation: two in HCP-identified hand knob area (area 6d), one in HCP-identified ventral premotor cortex (6v), and one on the border of the HCP-identified premotor eye fields (PEF) and speech-related 55b. Implant targets were guided by a multimodal cortical parcellation^53^ of the left precentral gyrus. T16 was able to speak slowly and quietly, but enunciation was restricted by limited face and mouth movement. She had limited voluntary control of her upper extremities, with some shoulder motion and some slow and contractured wrist and finger movements. She had limited to no voluntary control of her lower extremities. T16’s sensation was fully intact. Data are reported from post-implant days 88–377.

T17, a right handed man, was 33 years of age at the time of this study with an ALS diagnosis. In February 2024, six 64-channel 1.5 mm-length silicon micro electrode arrays coated with sputtered iridium oxide (Blackrock Microsystems, Salt lake City, UT) were implanted in T17’s left precentral gyrus, guided by individualized HCP cortical parcellation^53^: two in HCP-identified hand knob area (area 6d), two in HCP-identified ventral premotor cortex (6v), and two targeting HCP-identified area 55b. At the time of data collection T17 had incomplete locked-in syndrome. Specifically, T17 is anarthric, quadriplegic, and ventilator dependent; his only volitional motor control is his extraocular muscles, which he uses for communication (ALS-FRS score of 0 at the time of study enrollment). Data are reported from post-implant days 284–287.

No sample size considerations were performed given the investigative nature of the study; however, we focus our analyses on replicating findings across participants. The primary inclusion criteria for the study was participants ’clinical history and its congruence for an invasive neural implant. While we happen to enroll two participants of each sex, the study did not control for other socioeconomic, race, ethnicity, gender or a combination of these factors.

### METHOD DETAILS

#### Functional MRI Speech Lateralization & Array Placement

Prior to surgery, all participants underwent anatomic and functional brain imaging for speech and language lateralization, surgical planning and array placement targeting. (see ^1,2,75^ for array location estimates and further details).

#### Neural signal processing

Voltage time series signals were recorded using the Neuroplex-E system (Blackrock Microsystems) and transmitted via a cable attached to a percutaneous connector. Signals were analog filtered (4th order Butterworth with corners at 0.3 Hz to 7.5 kHz), digitized at 30 kHz (250 nV resolution) and fed into custom MATLAB or Python software (BRAND^76^) for digital filtering and feature extraction (more details below). To isolate signals relevant for estimation of neural ensemble activity^77^, voltage time series were digitally high-pass filtered (250Hz cutoff) non-causally on each electrode using either a 1ms (T15, T17) or 4ms (T12, T16) delay, and linear regression referencing (LRR)^78^ was applied. Electrode-specific thresholds and LRR filter coefficients were determined using data recorded from an initial diagnostic or rest block at the beginning of each session that was structured like the active instructed delay task described in 2.2

Next, two estimates of neural ensemble activity were computed for each electrode in either 10ms or 20ms bins. Threshold crossings were computed by counting the number of times the filtered voltage time series crossed an amplitude threshold set at either −3.5 or −4.5 times the standard deviation of the voltage signal. Default parameters for bin size and threshold varied between individual clinical trial site defaults. Spike band power was computed by taking the sum of squared voltages observed during each time bin. Threshold crossing rates and spike band power are estimates of local spiking activity and have been shown to be comparable to sorted single unit activity in terms of decoding performance and neural population structure^79–81^. For each block, within each electrode, mean threshold crossing rates and spike band power was subtracted from each sample to account for neural nonstationarities (drifts in mean firing rate) which could arise over the course of a session^63,64^. Threshold crossings and spike band power features were also normalized by dividing by their standard deviation for all analyses apart from the PSTHs (Figure 1D), to ensure that electrodes with large feature values did not overly influence the population-level results.

#### Data collection rig

Digital signal processing and feature extraction were performed on a dedicated computer. For T12 sessions prior to December 2024, Simulink Real-time was used for data processing and the Psychophysics Toolbox ^82^ in MATLAB was used to implement task software. An additional Windows computer controlled task starting and stopping and interfaced with the Neuroplex-E system. For T12 sessions starting in December, T15, T16, and T17, BRAND^76^ was used to implement modular, Python-based neural data processing and task software.

For a summary of all data collection sessions see Table S3.

### Representation of Verbal Behaviors in motor cortex (Figure 1)

#### Stimulus word selection

To investigate the representation of verbal behaviors in the motor cortex we selected a small set of single-syllable English words with non-overlapping phonemes. The limited number of words was necessary due to research session time constraints and the need for repetition to obtain a sufficient average neural representation for each word and perform statistical analyses. We chose words consisting of non-overlapping phonemes and of similar duration (see Table S2), so that distinguishability would be based on phonemic content rather than timing. Common English words were selected to ensure participants were well-rehearsed in their articulation prior to onset of dysarthria and thus may have a more accurate articulatory plan than nonsense or uncommon words. Additionally, the specific words were chosen to be maximally separable in articulatory space. Consonant phonemes in the same position across words differ by at least one feature (place of articulation, manner, voicing), and all places of articulation (except glottal), manner, and voicing types were represented. Vowels were also sampled across the vowel space, and included dipthongs to increase complexity in order to further separate words.

#### Isolated Verbal Behavior Instructed-Delay Task

For each research session investigating ‘isolated ’verbal behaviors (where each verbal behavior was tested separately in its own blocks), participants performed each verbal behavior in an instructed-delay task. In the “active” conditions, where participants either attempted to speak or imagined speaking, the task consisted of a “delay” period and an execution, or “go,” period. During the delay period, a text cue was presented above a red square indicating that no behavior should be performed. When the square turned green and the text cue disappeared, the participant began performing the desired verbal behavior. In the “passive” conditions (silent reading or listening) the trial design consisted only of go periods where the text appeared (for silent reading) or an audio file played a recording of the word being spoken (for listening), followed by intertrial intervals for the participant to return to baseline resting state between trials. Trials were grouped into individual experimental “blocks” by behavior, and several blocks of each behavior were alternated throughout a research session. The exact durations of each trial period for a given verbal behavior were determined based on individual participants' comfort and attention level. Timings and total number of trials by behavior are shown in Table S4.

#### Naive Bayes classification (Figure 1E,F)

Offline classification results (reported in Fig 1E,F) were generated using the following methodology. First, to reduce high-frequency noise, concatenated binned threshold crossing rates and spike band power features were smoothed using a 60 ms Gaussian kernel. Second, we performed a nested 10-fold cross-validation strategy to find the optimized starting time of a 500ms window of activity for decoding. This was done separately for each behavior for each participant array due to variations in the timing of neural modulation across behaviors and participants (evidenced in Figure 1D). For example, the neural modulation for T16’s attempted speech peaks between 2 and 4 seconds after the go cue onset, whereas her mimed and inner speech conditions peak much earlier after cue onset. To avoid biasing the decoding accuracies upwards from overfitting the window start time, we used a nested 10-fold cross-validation strategy for window optimization (Figure S6).

For each outer fold (row in Figure S6), the window start time was optimized on the training data using an inner 10-fold cross-validation to estimate decoding accuracy for each possible start time (using a Gaussian Naive Bayes classifier, as described in ^83^). The highest-performing start time was then selected and applied to the test set of the outer fold, ensuring that decoding performance was never evaluated on data used to select the start time. We then concatenated the test-set evaluated success vectors for each fold to calculate the accuracy and confidence intervals, which are reported in Figure 1E. Note that decoding results could be aggregated across different windows if the same start time was not selected for each fold. Confidence intervals were computed using the Clopper-Pearson method for binomial distributions applied to the boolean array indicating whether predicted classes were correct for all cross validated predictions. A mean decoding accuracy was considered “significant” if the lower bound of the confidence interval was above the chance value of 14.3%. We saved the most common optimal window across outer folds for each participant-array and behavior for use in additional analyses in Figure 2. These values are shown in Table S5, excluding array-behavior combinations that had no significant decoding. We chose a Gaussian Naive Bayes classifier because it is a simple method that effectively demonstrated strong neural tuning; however, more advanced methods could likely improve classification accuracy.

#### Control for duration of articulation tuning (Figure S1)

Neural tuning simply for the duration of words (“articulatory length”), rather than their phonemic or articulatory content, could result in significant decoding performance. In order to control for this, we assessed tuning to articulatory length using the same windows of neural activity used for decoding (2.3). We reasoned that if articulatory length had a strong influence on neural activity, then words with a greater difference in articulatory length should evoke more distinct neural activity patterns. We assessed this by testing for statistically significant linear relationships between pairwise articulatory length differences and neural distances. Articulatory length for each word was estimated using the audio duration of each word as generated by a text-to-speech (TTS) model (AWS Polly aws-cli/2.22.29, Table S2). Euclidean distance in neural state space between pairs of words was computed using the cross-validated distance estimator described in^83^ for windows listed in Table S5. To assess significance for each array and behavior, ordinary least squares (OLS) linear regression was used to model the articulatory length differences as a predictor of neural distances (including an intercept term), and a two tailed hypothesis test was performed to assess whether the model coefficient was significantly different from zero (alpha = 0.05) (Supp. Fig. 2B-C).

The same OLS analysis was used to assess whether any significant relationship exists between decoding accuracy and articulatory length tuning across all arrays and behaviors (Supp. Fig. 2D). Decoding accuracy was computed for the per-behavior-and-array mode of the optimal windows found in 2.3.

Additionally, decoding performance was assessed using a shorter 360 ms time window (Figure S1E), as opposed to the 500 ms time window used above, to test that results still hold even if the window is too short to contain silence periods for the shorter words. The same methods for decoding and significance testing described in 2.3 were used.

### Shared structure across verbal behaviors (Figure 2)

In Figure 2 we further analyzed the isolated verbal behavior task data. Binned threshold crossing rates and spike band power features were smoothed with a 60ms Gaussian kernel, then averaged within a 500 ms window chosen for each behavior based on the decoding optimization sweep done for Figure 1E (see Table S5 for exact windows). This process resulted in a 128 × 1 feature vector for each array, behavior, word and trial, which were further analyzed as described below. We denote these feature vectors with the notation $f_{w,i}$, where i indexes over trials and *w* indexes over words.

#### Cross-Validated Correlation Metric (Figure 2A,B)

Before estimating correlations between individual words (Figure 2A) or whole behaviors (Figure 2B), we first subtracted the mean feature vector across all words within each behavior, yielding mean-subtracted feature vectors ${\hat{f}}_{w,i}=f_{w,i}-\frac{1}{N}\sum_{w,i}f_{w,i}$, where N is the total number of all trials across all words. These vectors ${\hat{f}}_{w,i}$ were then used to estimate correlations between word pairs (Figure 2A) using a cross-validated method described in detail in^83^. Note that this bias-reduced estimator allows for a resulting value to be greater than 1 (or less than −1), particularly when the neural modulation is weak. Values exceeding 1 (or below −1) should be interpreted as evidence that the true correlation is near 1 (or −1).

The motivation for using a cross-validated estimator is that the sample estimate of the correlation is biased towards zero. To see this, let $f_{a}=\frac{1}{N}\sum_{i}{\hat{f}}_{w=a,i}$_,_ (the average feature vector for word *a*, where *N* is the number of trials) and $f_{b}=\frac{1}{N}\sum_{i}{\hat{f}}_{w=b,i}$. Then the sample correlation can be written as:$\frac{\left(f_{a}-{\bar{f}}_{a}\right)\cdot \left(f_{b}-{\bar{f}}_{b}\right)}{\left\|f_{a}-{\bar{f}}_{a}\right\|\left\|f_{b}-{\bar{f}}_{b}\right\|}$, which is biased towards zero in the presence of observation noise because the magnitude terms in the denominator are inflated by noise. The cross-validated estimator of correlation computes the magnitude terms using cross-validation in order to reduce bias.

To compute the correlations across whole behaviors and not individual word pairs (Figure 2B), we first created “behavior” vectors that contain one trial of neural modulation for all seven words.

That is, the behavior vector for trial i was defined as:$b_{i}={\left[{\hat{f}}_{1,i},{\hat{f}}_{2,i},\ldots ,{\hat{f}}_{7,i}\right]}^{T}$. The cross-validated correlation estimator was then applied directly to these (128*7) × 1 behavior vectors from two behaviors of interest to yield a single correlation value. This value describes how similar the neural modulation across all 7 words is between those two behaviors. Note, we excluded arrays and behaviors from this analysis for which the neural modulation was not significantly decode-able based on the decoding analysis depicted in Figure 1E.

#### Normalized Neural Distance (Figure 2D)

In Figure 2C, we estimated the average Euclidean distance in neural state space between all pairs of words within each behavioral condition separately, using the cross-validated distance estimator described in ^83^. Within each participant, we normalized the distances by dividing by the average distance of the attempted vocalized condition in order to directly compare the relative scales of the word representations across behaviors and arrays.

#### Principal Components Analysis Visualization (Figure 2C)

To better visualize the neural geometry of attempted and inner speech, we plotted projections of the data in the top 3 principal components as determined by Principal Components Analysis (PCA). First, for each word and behavior, neural activity was time-averaged and averaged across trials, yielding a 128 × 1 representation vector (for each 64-electrode array). That is, following the notation above, each word’s feature vector $f_{w}$ was computed by averaging over all trials for each word:$f_{w}=\frac{1}{N}\sum_{i}f_{w,i}$. We fit PCA across the columns of a 128 × 7 matrix made up of the 7 word vectors for the attempted speech behavior. We then projected and plotted all 7 word vectors from attempted vocalized, attempted mimed and an exemplary inner speech condition for each participant into the 3-dimensional space created by the top 3 principal components. The inner speech condition was chosen for each participant based on having the largest average neural distance, normalized to attempted speech, as shown in Figure 2D. We connected nearby words with lines to form “word rings” to better visualize the relative positions of each word (lines were drawn and colored consistently across behaviors), and rotated the viewpoint to best reveal the relationship between the three word rings shown.

### Online inner speech decoder (Figure 3)

#### Session Design

Real-time, self-paced inner speech decoding was evaluated in sessions t12.2023.11.28, t15.2024.03.03, t16.2024.04.29 for the 50-word vocabulary, and in sessions t15.2024.12.15, t16.2024.12.18 and t15.2025.01.05 for the 125,000-word vocabulary (reported in Fig. 3). Each session began with either a “diagnostic” or “rest” block, which was used to calculate threshold values and filters for online LRR to be used throughout the session for T12 and T16. For T15, filter and LRR parameters were recalculated after every experimental block.

Next, we collected “open-loop” blocks of sentences (1–5 blocks of 40–50 sentences per block) as training data, with no real-time decoder active. In 50-word vocabulary sessions, each sentence block was collected twice: once with the participant performing inner speech and once with attempted speech. In the 125,000-word vocabulary sessions, only training sentences with the participant performing inner speech were collected. A decoder was then trained using the inner speech training blocks collected that day, along with previously collected attempted speech data (see Table S7). The decoding and training methods are the same as those reported previously^1,2^. The RNN architecture includes a unique input layer for each dataset, allowing attempted speech data from earlier sessions to be included in training without negatively affecting inner speech performance. For the 50-word vocabulary session days, attempted speech blocks from the same day were not used to train the online inner speech decoder. Note, that since T15’s model employed online retraining ^2,84^, even the sentences decoded in real-time were involved in continually retraining the model throughout the session up to and including during the evaluation block for both the 50-word and 125,000-word vocabulary evaluations. T16’s 125,000-word vocabulary decoder also utilized online training in which the cued sentences were used as ground truth to retrain the model, but only after those sentences had been decoded online.T12’s and T16’s 50-word vocabulary decoders however did not utilize online retraining. Amount and type of training data are described in Table S7 for each individual decoder.

After initial decoder training, we collected preliminary closed-loop blocks to allow the rolling z-scoring algorithm time to properly account for any nonstationarities that may have accrued during training. For T15 these additional blocks also served as training data, as the model was retraining online as described in ^2,84^. T16 had online retraining only for the 125,000-word vocabulary evaluation blocks, in which the cued sentences were used as ground truth to retrain the model, but only after those sentences had been decoded online. T12 did not have online retraining. Finally, a closed-loop evaluation block was collected. For the 50-word vocabulary sessions this evaluation set consisted of the 50 sentences from ^18^, also used for evaluation in ^1,2^. For the 125,000-word vocabulary evaluation block, we used a random selection of sentences from the Switchboard corpus similar to previous evaluation procedures in^1,2^. No evaluation sentences appeared in any of the training blocks.

#### Participant Inner Speech Behavior Strategy

For real-time inner speech decoding, we allowed participants to select their preferred form of inner speech they felt they could perform most consistently amongst the three inner speech behaviors tested in the isolated verbal behavior experiments described in section 2.2 and Table S1. When using the inner speech BCI, T12 performed Motor Inner Speech, T16 performed 1st Person Auditory Inner Speech, and T15 performed Motor Inner Speech for the 50-word evaluation in t15.2024.03.03. However, for the large vocabulary evaluation session t15.2025.12.15 participant T15 was initially cued to perform Motoric Inner Speech and afterward reported using a hybrid strategy of Motoric Inner Speech and Imagined Listening. Specifically, he imagined moving his articulators to produce the words and the sound of a well-known actor’s voice coming out. For consistency, we instructed him to perform the same strategy for the next session t15.2025.01.05.

### Vocabulary and Sentence Selection

In the 50-word vocabulary evaluation sessions, all of the inner speech sentences were constructed out of the 50-word vocabulary originally published in^18^ and the same evaluation sentence set was also used. There were no overlapping sentences between the training and evaluation sets. Previously collected inner speech data used to supplement the training were taken from the large vocabulary Switchboard corpus as described in^1,2^.

The sentence sets used for the 125,000-word vocabulary evaluation sessions were taken from the Switchboard corpus and the vocabulary was taken from the CMU pronouncing dictionary, as described in^1^. There were no overlapping sentences between the training and evaluation sets.

Previously collected attempted speech data used to supplement the training used sentences was also taken from the Switchboard corpus. For participant T15, correctly decoded sentences from previous personal-use of the speech BCI were also used to supplement training. The number of evaluation sentences used for each session were the following: t15.2024.12.15 (15), t16.2024.12.18 (29) and t15.2025.01.05 (25).

#### Real-time Decoder Training Data

To assist in training the online decoder for inner speech, we also incorporated previously collected attempted speech data (vocalized or mimed). The RNN architecture included a separate input transformation layer for each included session that was trained from scratch as described in^1^. The total number and type of sentences used to train the models which were evaluated in real-time are described below. Note, that since T15’s model employed online retraining ^2,84^, even the sentences decoded in real-time were involved in continually retraining the model throughout the session up to and including during the evaluation block for both the 50-word and 125,000-word vocabulary evaluations. T16’s 125,000-word vocabulary decoder also utilized online training in which the cued sentences were used as ground truth to retrain the model, but only after those sentences had been decoded online.T12’s and T16’s 50-word vocabulary decoders however did not utilize online retraining. Amount and type of training data are described in Table S7 for each individual decoder.

#### RNN

To transform neural activity evoked by inner speech into a time series of phoneme probabilities, we used a 5-layer, stacked gated recurrent unit RNN as described in^1^. RNN parameters for T12 were determined based on the results of^1^ and for T15 from^2^. For T16, parameters optimized for mimed speech were used. To train the model without any ground truth timing labels (given the participants ’inability to produce intelligible speech) we used a connectionist temporal classification (CTC) loss. We also added two types of artificial noise to help regularize the model. For details about training methods see^1^.

#### Language Model

We employed an n-gram language model (LM) to decode word sequences from RNN outputs for both real-time and offline analyses. First, we built the n-gram LM with Kaldi^85^ using the OpenWebText2 corpus ^86^. We reprocessed the text to retain only English letters and a limited set of punctuation marks. Next, we used Kaldi to construct the n-gram LM either with the CMU Pronunciation Dictionary (http://www.speech.cs.cmu.edu/cgi-bin/cmudict) (125,000 words) or a 50-word vocabulary from ^18^. The resulting LM was represented as a weighted finite-state transducer ^87^, allowing us to map sequences of CTC labels to candidate sentences in real-time. This followed the same procedure detailed in ^1^. Additionally, for T15’s online speech decoding a transformer LM ^88^ was used to rescore the candidate sentences in a third pass to further improve decoding accuracy at the end of each sentence trial. For this, we used the publicly available pretrained OPT LM ^89^. For additional details on language model parameter selection and inference see ^1,2^.

#### Word Error Rate

We evaluated decoding performance using word error rate (WER), defined as the edit distance between the decoded word sequence and the target prompt sentence—that is, the number of insertions, deletions, and substitutions required to make the sequences match exactly. WER can exceed 100% when the total number of errors surpasses the number of words in the original prompt.

All reported error rates are aggregate WERs, computed across many independent sentences. To calculate this, we summed the total number of errors across all sentences and divided by the total number of words in the corresponding reference sentences. This method avoids over-weighting very short sentences, which could disproportionately affect sentence-level averages. Confidence intervals for WER were estimated using bootstrap resampling over individual trials, recomputing the aggregate WER across 10,000 resampled datasets.

To estimate chance performance, we randomly permuted the ground truth labels with respect to the decoder outputs, so that decoder outputs no longer corresponded to the correct target sentence. The aggregate WER was computed across all evaluation sentences for each shuffled set (10,000 iterations). The chance level was defined as the lower bound of the bootstrapped 95% confidence interval from this shuffled distribution. Note that this method of estimating chance performance only tests whether a significant relationship exists between the decoder outputs and target sentences, and can yield chance levels greater than a 100% word error rate, which is worse than the performance that could be achieved by optimally guessing (for example, outputting nothing). This is because shuffling only assesses whether the decoder output is related to the target; for example, if the decoder outputs the correct sentence repeated 5 times, then the word error rate will be 400% (since all extra words must be deleted for the output to match the target), but chance level will be even higher (because shuffling will require nearly *all* words to deleted or changed, not just those that are repeated).

### Inner speech as a mental strategy for serial recall in upperextremity motor sequence tasks (Figure 4, Supp. Figure 4, 5)

#### Upper-extremity motor tasks without mental strategy instructions

Three tasks were designed to variably elicit verbal or non-verbal short-term memory for the execution of an upper-extremity motor task, with the hypothesis that representations of inner speech for cognition could be decodable from speech-motor areas. In a suite of instructed-delay tasks, sequences of upper-extremity movement directions were cued and subsequently executed during the go period. Removal of the visual sequence cue during the go period enforced the use of short-term memory to execute the cued movement sequence. All tasks were described to the participants as an upper-extremity motor task without any explicit instruction about what kind of mental strategy to employ. The 3-element arrows task consisted of sequences of three arrows pointing in one of four directions (↑ → ↓ ←) as well as a ‘Do Nothing’ cue. All possible sequences were used, resulting in 65 conditions. A subset of these conditions using only two directions (↑, →) were used for T16 due to session time constraints. T12 was instructed to sequentially move a joystick in the directions of the displayed arrows, returning to center between sequence positions. The single-element arrows task was identical except that only a single movement direction was cued. This task was not done with T16 due to session time constraints. The 3-element lines task was cued with an image of line segments which participants were instructed to reproduce by drawing. The displayed image showed the starting point and three line segments indicating the three target movements. After an audible go cue, the image was removed after which participants attempted to reproduce the previously displayed image. After a go period, there was a 1.5 second return period to allow T12 to return the pen tip to the start location. Because T12 retained some control of her arm and hand, ground truth joystick and pen tip trajectories were recorded (see 7.1.4). Due to a greater degree of paralysis, T16 was only able to attempt to do hand movements for these tasks. Participant-specific task parameters and trial counts are reported in Table S4.

#### Upper extremity motor tasks with instructed mental strategy

For the instructed verbal memory and visual memory upper-extremity motor tasks, the same task design was used but with different instructions for delay and go period mental strategies. Cues consisted of sequences of three arrows pointing either up (↑) or right (→). During the go period the arrows were removed and participants were instructed to attempt to draw a sequence of line segments in the direction of the arrows similar to the 3-element lines. Pen tip trajectories were recorded during drawing to assess recall accuracy. For verbal memory tasks, participants were instructed to use inner speech as a mental strategy for short-term memorization of the cued arrow sequence. For visual memory tasks participants were instructed to use visual short-term memory and to suppress any inner speech about arrow directions. Participants were given time to practice until they felt comfortable with the task and reported being able to reliably engage each mental strategy. Participant-specific task parameters and trial counts are reported in Table S4.

#### Analogous attempted speech sequence recall task

The previously described verbal memory task was compared with a speaking task in which T12 was presented with the same direction sequences but via a recorded audio cue. The behavioral instruction was to attempt to speak the directions. Due to limited session time, T16 performed a smaller set of conditions consisting of a single direction (either “up” or “right”) rather than sequences of three directions, and cues were presented as text. For mental strategy, T12 was instructed not to change how she would naturally recall and speak the audio-cued direction sequence. Participant-specific task parameters and trial counts are reported in Table S4.

#### Hand movement tracking

For tasks requiring joystick movement (3-element arrows, single element-arrow), T12’s hand movement was recorded using a Logitech Extreme 3D Pro Gaming Joystick. For drawing tasks (3-element lines, inner speech, no inner speech), T12 was instructed to draw on a 15 inch LCD writing tablet (ERUW Shenzhen Lei Rui Technology Co., Ltd) using a stylus. Lines were erased between blocks. The starting position was indicated by drawing an X on the tablet before each block. The Optitrack v120 Trio was used to track the three dimensional positions of the stylus and writing tablet. Six infrared reflective markers on a modified Optitrack Hand Rigid Bodies Marker Set were attached to the back of the stylus using a custom 3d printed mount. Six additional infrared reflective markers were affixed to two adjacent sides of the writing tablet which faced the Optitrack camera using Optitrack Marker Bases in order to estimate the writing plane. Both the stylus and the writing surface were recorded in real time as custom rigid bodies with the three dimensional coordinates of all infrared markers recorded using Motive. The trajectory of the stylus tip on the trackpad was estimated by using the stylus location and orientation as well as the 2d writing surface. At each time sample the stylus rigid body was represented as a quaternion. Tip location was estimated from the recorded quaternion by subtracting the quaternion of a reference stylus with measured tip location. The writing plane was estimated from selecting three points from the tablet markers. Finally, the stylus tip location within the 2 dimensional coordinates of the writing plane was estimated by rotating the stylus quaternion along the writing plane’s normal vector.

The inferred stylus-tip trajectories were used to assess sequence recall accuracy. Individual trial pen tip trajectories were visually assessed and compared to the instructed cue. Zero errors were made by T12 during the verbal and visual memory tasks.

No ground truth motor activity could be collected with T16 due to a higher degree of paralysis limiting her ability to draw.

#### Using binary decoding to assess sequence encoding per position

Threshold crossing rates were summed over a 2 second window before the go cue to isolate preparatory activity, generating a 64 length vector for each trial and microelectrode array. Decodability of individual positions was estimated by comparing two sequences that differ in only a single position. For T16, a 0.75 second window immediately following the go cue was used. To assess the neural encoding for a movement direction in only one sequence position, binary LDA decoders^90^ were fit for each pair of sequences that differed in only one position. Assessing decoding performance of individual positions, with the other elements of the sequence held constant, helps to reduce potential confounding effects of sequence context that could artificially lower performance of a decoder which classifies across all possible contexts together. Binary decoders were fit to classify between pairs of conditions in order to compare performance between tasks that varied in the number of possible sequence elements in each position. Five-fold cross-validation was used to prevent overfitting. Boxplots for each sequence position depict the distribution of decoding accuracies across all possible pairs differing at that position (Figure 4).

#### Confidence interval estimation via bootstrap

Confidence intervals for decoding performance at each sequence position were calculated by resampling decoder predictions. For each decoder, the cross-validated predictions were resampled with replacement. Resampled predictions from all decoders for a specific sequence position were joined to estimate the per-position decoding accuracy. This was repeated 10,000 times to estimate the distribution of per-position decoding accuracy. Significance was assessed by taking the 0.025th quantile and comparing it to the null hypothesis of chance-level decoding accuracy (0.5).

#### Paired increase in decoding accuracy for instructed verbal vs visual memory task

To assess the significance of the effect of explicit instruction to use either verbal or visual memory for sequence recall, the distribution of increases in decoding accuracy for paired decoders differing only in instructed mental strategy was estimated per position. For each decoder, the cross validated predictions were resampled with replacement to estimate per-decoder resampled accuracy. Then, paired decoding accuracies for data only differing in instructed mental strategy were subtracted in order to compute the increase in decoding accuracy due to instruction to use a verbal versus visual mental strategy. Finally, this was repeated 10000 times to estimate the distribution of increased decoding accuracy for each position. Significance was assessed by taking the 0.025th quantile and comparing it to the null hypothesis of no increase in decoding accuracy.

#### Cross task positional decoding

To assess whether sequence representation in the hand-motor task was indeed due to inner speech, we tested whether decoders trained on a speech sequence task could generalize to the hand-motor task when inner speech is used. LDA decoders were fit similarly to 5.2 except that train and test data were from different tasks.

### Uninstructed inner speech in the conjunctive counting task (Figure 5)

To further explore non-speech tasks that may naturally engage uninstructed inner speech, we asked participants to complete a conjunctive counting task with the hypothesis that participants would use inner speech to sequentially count targets and that this inner speech would be able to be decoded by an RNN trained to decode instructed attempted speech. This task was conducted with T15 and T16 in experimental sessions t15.2024.12.15 and t16.2024.12.18.

#### Task description

We designed visual stimuli similar to the conjunctive visual search paradigm^91^. An image of a 10 by 10 grid of colored shapes was presented to participants while neural data was recorded. Two shapes and two colors were selected randomly from a set of 9 colorblind-friendly colors and 6 distinct shapes, resulting in four distinct objects per image (Figure 5A). Above the image, participants were prompted to count all appearances of one shape-color combination, of which there were in total between 10 and 20 appearances. We hypothesized that the non-target objects in the grid would act as visual distractors, thereby encouraging participants to rely on an inner speech sequential counting strategy to accurately tally the target items. Participants pressed a button to signal the end of the counting phase, which then triggered the transition to the reporting epoch during which they attempted to speak the final counted number. Then, after another button press, the trial progressed to a confirmation epoch in which participants were asked whether they said the correct final count (yes/no).

#### Large-vocabulary inner speech sentence task control

To test the null hypothesis that increasing sequences of numbers would be decoded by chance, resulting in a spuriously positive regression slope, we performed the same number-word decoding analysis on trials from the instructed inner speech large-vocabulary sentence training data to generate a plausible null distribution. Due to the difference in duration between the conjunctive counting trials and the sentence trials, we randomly combined 3 sentence trials to create control trials with an average duration matching that of the counting trials. We then performed the same offline decoding and regression analysis (including using the same RNN and language model). To generate a distribution of chance slopes, we ran 1,000 resamplings of N stitched-together sentence trials (where N represents the number of counting trials for each participant, and trials were stitched together differently each resampling) and plotted the histogram of the resulting slopes. These were compared to the slope from the regression line of the counting task analysis (Figure 6G-H).

### Verbal and autobiographical thought prompts (Figure S5)

To further explore free-form inner speech, we asked participants to engage in a variety of thought patterns. Participants were prompted via text to either engage in verbal thought (concrete sequences of words e.g. “Think about the lyrics of the first song that comes to mind.”) or to engage in autobiographical thought (e.g. “Think about your daily morning routine”). The full text of all prompts is listed in Figure S5. All trials were self paced, allowing participants to take as much time as needed to complete the thought prompt. No instruction about specific mental strategies was given. Each prompt was presented once, except for “clear your mind” which was presented 10 times. All prompt categories were interleaved.

We hypothesized that participants would engage in inner speech during the verbal thought prompts which could then be decoded by an RNN trained on instructed inner speech. We hypothesized that the number of decoded words during verbal thought prompts would be greater than during the “clear your mind” trials, indicating that free-form naturalistic inner speech could be decoded by a speech BCI trained on instructed inner speech.

We also included autobiographical thought prompts, chosen for the potential for participants to engage other forms of thought distinct from verbal thought (e.g., visual imagery). Participants were not explicitly instructed to avoid engaging in inner speech during the autobiographical thought prompts, so it is possible that participants could have used inner speech (for example, accomplishing the prompt “think about your daily morning routine” by internally verbally describing it). It is also possible to accomplish the autobiographical prompts by engaging in other forms of thought such as episodic memory or sensory imagery (e.g. visual, auditory, olfactory). Therefore, we hypothesized that the number of words decoded during verbal thought would be greater than or equal to (but not less than) the number of words decoded during the autobiographical prompts.

To assess the number of decoded words per trial, the same large-vocabulary RNN and language model were used as described in above sections, except that the analysis was performed offline. Due to the uncertainty of whether the decoded text is representative of the participants ’thoughts, and due to mental privacy concerns, we elected not to include the decoder output for this task in the manuscript. 95% confidence intervals for the number of decoded words per category were computed via bootstrap resampling of trials 10,000 times.

To ensure that any difference in number of decoded words was not due to differences in trial duration, since the task was self-paced, we also plot the mean and 95% confidence interval of trial duration by cue type (Figure S5C), (computed by bootstrap resampling of trials 10,000 times).

### Relationship between attempted and speech conditions (Figure 6)

While the ‘isolated ’verbal behavior experiments (section 3) allowed us to explore a large number of behaviors, it did not allow us to assess differences in neural representation between behaviors, because any differences between blocks could also be caused by spurious neural nonstationarity known to be occur in microelectrode array recordings^63,64^. In order to assess whether differences exist between the neural representation of attempted and inner speech (which could be a useful cue for a decoder to distinguish between them), we ran a follow-up task in which attempted speech, inner speech, and listening conditions were randomly interleaved within an experimental block. When trials are interleaved within a block, any differences in mean between behaviors is preserved when performing block-wise mean subtraction to remove firing rate drift across time.

#### Interleaved Verbal Behavior Instructed-Delay Task

This task included three behaviors randomly interleaved within the same experimental block (attempted vocalized speech, motoric inner speech, and listening for T12; attempted vocalized speech, 3rd person auditory inner speech and listening for T15; attempted mimed speech, 1st person auditory inner speech, and listening for T16; attempted speech, 3rd person auditory inner speech, and listening for T17). Participant-specific trial counts are reported in Table S4.

#### Principal Component Analysis Visualization (Figure 6A-D)

To further compare the neural geometry of inner and attempted speech, we plotted projections of the data in the top 3 principal components as determined by Principal Components Analysis (PCA). First, for each word and behavior, neural activity was time-averaged and averaged across trials, yielding a 128 × 1 representation vector (for each 64-electrode array). We fit PCA across the columns of a 128 × 14 matrix made up of the 14 word-behavior conditions (7 words × 2 behaviors). We then projected and plotted all 14 average word vectors into the 3-dimensional space created by the top 3 principal components. We connected nearby words with lines to form “word rings” within each behavior to better visualize the relative positions of each word (lines were drawn and colored consistently across behaviors), and rotated the viewpoint to reveal angles for which shared structure between the rings and separation between the rings can be observed.

#### Cross-validated Euclidean neural distance within and across behaviors (Figure 6E)

In Figure 6E, we estimated the average Euclidean distance in neural state space between all 21 word pairs within a behavior (“within inner speech” and “within attempted speech” bars) and between all 7 matching word pairs across behaviors (“Motor-intent Dim.” bars) following the methods described in ^83^. Across-behavior distances estimate the size of the change in mean neural features between the behaviors (which together constitute a “motor-intent dimension” in neural state space), while within-behavior distances estimate the size of the neural modulation evoked by words. Confidence intervals (95%) were estimated for the mean within-behavior distances (21 points) and across-behavior distances (7 points) by assuming the points were normally distributed.

#### Motor-intent dimension definition and removal (Figure 6F-G)

To quantify the offset shift we observed in the attempted speech vs. inner speech word rings visualized in Figure 6A-D, we operationalized a “motor-intent dimension” defined as the direction of a vector connecting the centroids of the attempted and inner speech conditions.

First, we averaged across the repetitions of individual words within each behavior. Let:

- $x_{j,w}^{is}$ denote the feature vector from j the th trial in the inner speech (i.s.) condition for word condition w (with w=1,….7)
- $x_{i,w}^{as}$ denote the feature vector from the 𝑗th trial in the attempted speech (a.s.) condition for word condition w (with w=1,….7)
- $x_{j,w}^{is}$ and $x_{i,w}^{as}$ have dimensionality N × 1, where N is the number of neural features (N=128 for each array, composed of 64 threshold crossing features and 64 spike band power features). For each behavior, all trials and all word conditions were used to estimate the centroids. Formally, the centroids for the inner speech and attempted speech conditions are given by:

$$c_{is}=\frac{1}{7N_{is}}\sum_{w=1}^{7}\sum_{j=1}^{N_{is}}x_{j,w}^{is}\;\text{and}\;c_{as}=\frac{1}{7N_{as}}\sum_{w=1}^{7}\sum_{j=1}^{N_{as}}x_{j,w}^{as}$$

Here $N_{is}$ and $N_{as}$ represent the number of trials for the inner speech and attempted speech conditions, respectively.

The motor-intent dimension is defined as the normalized vector difference between the attempted speech and inner speech centroids:

$$v_{motor‐intent}=\frac{c_{as}-c_{is}}{\left|\;\left|c_{as}-c_{is}\right|\;\right|}$$

This direction vector captures the axis along which the word rings appear to shift from inner speech to attempted speech (Figure 6A-D).

To remove the influence of the motor-intent dimension from the neural data, we simply subtract the projection onto the motor-intent dimension from each original feature vector. The residual vector, which is orthogonal to $v_{motor‐intent}$, is given by:

$$x_{res}=x-\left(x\cdot v_{motor‐intent}\right)v_{motor‐intent}$$

### Offline self-paced inner speech analysis (Figure 3E, Figure 7A-D)

#### Attempted and Inner Speech Training and Test Sets

To further probe the relationship of attempted and inner speech in the context of self-paced sentences, we collected both attempted and inner speech datasets consisting of an identical sentence set. Next, we evaluated decoders offline using train / test splits of those sentences that were identical across behaviors. All sentences were constructed from the 50-word vocabulary. If a sentence was removed due to an interruption during data collection, the corresponding sentence in the other behavior was also removed. Interruptions during data collection that warranted trial removal consisted of coughing bouts, participant care needs, interruption by person in the room, or loud noises that masked the sound of the task computer, (i.e. from a passing train), all of which we believe prevented the participant from being able to fully perform the task during the given trial.

#### Offline decoding evaluation (Figure 3E)

To compare decoding performance directly between attempted speech and inner speech (Figure 3E), we trained offline models using only the self-paced 50-word sentences collected on the 50-word inner speech decoding evaluation day for T12, T15 and T16. We trained 10 model seeds each for attempted and inner speech datasets, and evaluated each model on the corresponding behavior’s test set, yielding directly comparable performance numbers for attempted and inner speech. The test sets consisted of the final 30 sentences collected for each participant.

Next to further assess the extent of the shared neural code between attempted and inner speech in the context of self-paced sentences, we evaluated cross-decoding performance (i.e., how well a decoder trained on attempted speech could decode inner speech). We did this by evaluating the attempted models on the inner speech test sets. RNN hyperparameters for all offline analyses were taken from our prior work ^1,2^.

To estimate a baseline (chance-level) word error rate—what you might expect if the RNN produced sentences that were unrelated to the target output—we shuffled the decoded sentences relative to their corresponding ground truth sentences. This shuffling was repeated 1,000 times for each of the 10 seeds in each scenario: (1) trained and tested on attempted speech only, (2) trained and tested on inner speech only, and (3) trained on attempted speech but tested on inner speech. In every scenario, the confidence intervals for the actual word error rates were significantly lower than the chance-level estimates.

#### “Imagery silenced” vs. “Imagery naive” training strategies

We defined two training strategies for RNN decoders in speech BCIs that decode neural activity into sequences of phoneme probabilities. The “imagery naive” strategy—commonly used in previous studies—labels trials of neural activity from attempted speech sentences with their corresponding phonemes. In contrast, our proposed “imagery silenced” strategy incorporates both attempted and inner speech data. In this approach, trials from attempted speech are still labeled by their phonemes, while trials from inner speech are labeled solely with the silence token “SIL.”

#### Decoding performance by training strategy (Figure 7A-B)

To assess the performance of the imagery-silenced and imagery-naive training strategies, we first measured the word error rate of RNNs trained using each method on attempted speech evaluation trials (the target output, Figure 7A). This analysis followed the same procedure described in section 4.7. Next, we evaluated the frequency at which RNNs trained by each method erroneously decoded inner speech trials (i.e. trials where output decoding should be avoided, Figure 7B). Here, any trial where a token other than the “SIL” silence token was produced by the RNN was considered a failure. 95% confidence intervals were calculated in a similar bootstrap manner as described in section 4.7 for WER in which 1,000 resamplings were taken for each of the 10 model seeds. These 10,000 samples were used to compute the confidence interval (2.5 to 97.5 percentiles).

#### Correlation of logits (Figure 7C)

To assess the effect of the "imagery-silenced" training strategy on decoder output, we analyzed the phoneme probabilities (logits) given by the RNN decoder for paired attempted and inner speech trials. We reasoned that in the imagery-naive case, the decoder might inadvertently output similar probability profiles for matched attempted and inner speech trials due to the high correlation between attempted and inner speech. In contrast, the imagery-silenced strategy should ideally yield less similar outputs. To quantify this, we first time-warped the decoder outputs to align the paired attempted and inner speech trials (thereby accounting for natural variation in speaking rate), and then computed the correlation (Pearson’s r) between the time warped logit time series.

In order to align the attempted and inner speech trials (which might have similar content but be misaligned in time due to different rates of speech, and typical behavioral variation across trials), we used dynamic time warping ^92^. To do this, we used the python dynamic time warping package ^92^ with a ‘symmetric2 ’step pattern and slanted band window of a 100ms size. This allows for some flexible time alignment while also constraining variation so that time-warping cannot be too extreme and overfit to noise.

After aligning corresponding attempted and inner speech logit time series with Dynamic Time Warping, we calculated the correlation (Pearson’s r) for each sentence separately. For each sentence, we computed a correlation for all 39 phoneme logits, and then averaged them to yield a single value. This procedure was repeated for all 30 test sentences and 10 decoder seeds for each training strategy. The bar heights in Figure 7C represent the average across all 30 sentences and 10 seeds. To estimate chance levels, we shuffled sentences within each behavior, such that sentences were no longer paired when correlating. The shuffling was repeated 1,000 times for each of the 10 seeds, and the mean of the resulting distribution was used as the chance value (dashed line). Confidence intervals (95%) were obtained via bootstrap resampling and then re-computing the average correlations over the resampled distribution (10,000 resamples).

### Real-time Evaluation of an Inner Speech BCI with Keyword Detection (Figure 7)

To prevent unintentional decoder output when using an inner speech BCI, we investigated a "keyword" strategy in which decoder output is turned on only when a special keyword is detected. A phonetically complex keyword that would rarely be spoken otherwise (“chittychittybangbang”) was chosen to increase the specificity of keyword-detection. T12 was presented with sentences from a 50 word vocabulary and instructed to internally speak them using a motoric inner speech strategy, using an instructed-delay task design described above except with an added * symbol in front of randomly chosen sentences. This * symbol was a cue to internally speak the keyword before internally speaking the cued sentence.

We used the speech decoding pipeline previously described. An RNN decoder was trained to predict sequences of phonemes, including all trials with and without keywords. Trials that begin with the keyword cue were labelled with the phoneme sequence of the keyword appended to the beginning of the trial. For example, “ * I need good music” was labelled as ['CH','IH','T','IY','CH','IH','T','IY','B','AE','NG','B','AE','NG','SIĽ,’AY’,’SIL’,’N’,’IY’,’D’,’SIL’,’G’,’UH’,’D ’,’SIL’,’M’,’Y’,’UW’,’Z’,’IH’,’K’,’SIL’].

Recently collected speech data from other research sessions was also included to aid in RNN training, including tasks for other research aims (See Table S7 for details).

A customized language model was built (as described in section 4.6) that included the 50-word vocabulary as well as the chosen keyword, “chittychittybangbang”. The training corpus included both the original 50-word vocabulary corpus as well as a duplicated version of it, in which all sentences had “chittychittybangbang” appended to the front. This ensured that language model statistics for the likelihood of detecting the keyword at the start of utterances would be equal to that of not predicting the keyword, and matched the distribution of the training and evaluation cue sets. Lastly, logic was added to the real-time decoder to suppress all output unless the word “chittychittybangbang” was detected by the language model.

To evaluate the real-time decoder with keyword detection in session t12.2024.12.19, we collected 80 trials consisting of the same 40 sentence cues repeated with and without the keyword. A trial was considered successful if a trial cued with a “*“ resulted in decoder output, or if a trial not cued with “*” did not output anything. We reported the mean success rate (binary) and calculated the 95% confidence interval using bootstrap resampling over individual trials (10,000 resamples). Additionally, the word error rate for keyword trials in which the decoder correctly produced words was calculated as previously described. The 95% confidence intervals for these error rates were also estimated via bootstrap resampling over individual trials and then re-computing the aggregate error rates over the resampled distribution (10,000 resamples).

### QUANTIFICATION AND STATISTICAL ANALYSIS

Analyses and statistics were performed using custom MATLAB and python code that is publicly available (see Data and Code Availability) and are described in detail in the STAR methods. Summary statistics, sample details, error bar details, and hypothesis tests are described for every figure in Table S6.

### ADDITIONAL RESOURCES

Clinical trial registry number NCT00912041

(https://clinicaltrials.gov/study/NCT00912041?id=NCT00912041)

## Supplementary Material

1 Figure S1: Neural tuning for word duration (“articulatory length”) does not explain separability between words or the 7-word decoding performance in Figure 1, Related to Figure 1
A) Diagram of per-array analysis to estimate tuning to articulatory length. Text-to-speech models were used to generate audio for individual words. Pairwise differences between word audio durations were regressed against pairwise differences in neural distance using ordinary least squares regression. If articulatory length is encoded in neural population activity, neural differences would be explained by differences in articulatory length. B) Articulatory length tuning was computed for each array and speech behavior, matching Figure 1E. Black asterisks indicate significant tuning for articulatory length (ordinary least squares regression coefficient p-value < 0.05). Red X's indicate arrays that do not have significant decoding of seven words reported in Figure 1E. Most arrays with significant decoder performance do not significantly encode articulatory length. C) Two example scatter plots of differences for each word pair in neural distance and articulatory length shown for the highest decoding performance array and behavior (T12 i6v Attempted, red) and highest articulatory tuning array and behavior (T15 i6v Listening, blue, p-value=5.68 × 10^-5) D) Although some behaviors in some arrays do have significant tuning for articulatory length, no significant relationship between articulatory length and decoding accuracy was found across all behaviors on all arrays (p-value=0.25). E) As an additional control, the decoding analyses from Figure 1E were replicated using a shorter window of neural data matching the shortest word duration (were, 366ms). Limiting the analysis to a window aligned with the shortest word should help reduce the possibility that activity related simply to the presence vs. absence of speech contributes to word classification. Decoding results were broadly similar. This indicates that discriminability between words across behaviors and arrays was likely due to neural tuning for phonemic or articulatory variation across words.

2 Figure S2: Shared representation of spoken direction words and verbal memory of a motor sequence‥ Related to Figure 4
A) An attempted speaking task was compared to a motor sequence task where participant T12 was instructed to use verbal memory to remember the sequence. B) The same decoding analysis described in Figure 4B is shown for decoders trained on attempted speech and tested on verbal memory (or vice versa) to assess whether the representation of verbal short term memory and spoken direction words is shared. Box plots show cross-validated accuracy (dotted line indicates chance) and asterisks indicate above chance performance per position as assessed via a bootstrap-derived 95% CIs compared to chance level of 0.5.

3 Figure S3: Inner speech during task execution is also decodable in i6v in T16, whereas areas 6d in T16 and s6v in T12 exhibit hand-motor tuning. Related to Figure 4.
A) T16 also completed the 3-element arrows and lines tasks without explicit instruction for mental strategy. Due to T16's upper-extremity paralysis, attempted drawing was instructed as the desired behavior for sequence recall (as opposed to actual drawing in participant T12). Task design for T12 is described in Figure 4A. **B)** Decodability of sequence position was assessed as in Figure 4. For T16, a window of neural activity from the first 1.5 seconds after the go cue was used to fit decoders. For T12 the same 2-second delay period window was used as reported in Figure 4. For area i6v in T16, only the 3-element arrow task elicited significant neural representation of the first sequence position. Our prior work^75^ has shown that area 6d in T16 and area s6v in T12 both encode hand-motor activity. In line with this, decoding performance was similar across all tasks, particularly for the first sequence position for T12-s6v which is distinct from T12-i6v results reported in Figure 4. Therefore, decoding results from these regions serve as a demonstration of the validity of the motor and sequencing control tasks (single-element arrow, and 3-element lines). **C)** T16 performed two versions of the three-element arrows task, but with explicit instruction to either use or suppress inner speech for short-term memory of the arrow sequence. **D)** Same as B but for tasks that only differed in instructed mental strategy. Instruction to use verbal mental strategy significantly increased decoding accuracy of the first position in area i6v in T16 (mean decoding accuracy 0.61, 95% CI 0.53–0.68) but not in areas 6d in T16 nor area s6v in T12. **E)** T16 was visually cued by text to speak a direction to test whether verbal short term memory in i6v had a shared representation with attempted speech. **F)** Same as B except decoders are trained on attempted speech (verbal memory) and tested on verbal memory (attempted speech). For T16 i6v, only attempted speech → verbal memory decoders could generalize above chance for the first position. Decoders did not generalize well for area 6d in T16.

4 Figure S4: Number words were more likely to be decoded during counting by a large vocabulary inner-speech BCI as compared to sentences. Related to Figure 5.
Frequency of numbers decoded offline during the conjunctive-counting task (blue) compared to real-time decoded inner speech sentences (orange), which were drawn from the Switchboard corpus. For the counting task, decoding was performed offline using the same RNN and language model as in real-time decoding using a 125,000-word vocabulary (Figure 3). Unlike the analysis done in Figure 5, this involved using our standard, large-vocabulary 5-gram language model that could decode non-number words as well as numbers. As expected, numbers were decoded significantly more often from neural data recorded during the conjunctive-counting task (T15: 0.45, 95% CI [0.2, 0.75]; T16: 2.0, 95% CI [0.85,3.45]) as opposed to the instructed inner speech Switchboard sentences task (T15: 0.0, 95%CI [0.0,0.0]; T16: 0.09, 95% CI [0.0,0.2]) - with significance determined by non-overlapping confidence intervals), which further supports the conclusion that uninstructed inner speech, such as that elicited during counting, can be decoded by a speech BCI. Confidence intervals were computed via bootstrap resampling (10,000 resamplings).

5 Figure S5: Offline decoding of neural activity recorded during prompted verbal and autobiographical thought shows that more words were decoded during verbal prompts compared to “clear your mind” prompts. Related to Figure 5.
A) Participants T15 and T16 engaged in a series of verbal or autobiographical thought prompts presented as text on a computer monitor. Participants were sometimes also prompted to “clear your mind”. We hypothesized that participants would engage in free-form inner speech during the verbal prompts, which would be able to be decoded by an RNN trained on instructed inner speech. Autobiographical prompts in which thought process could have taken on any different number of modalities (i.e. episodic memory or visual imagery, abstract representations, or inner speech) were also investigated. **B)** Number of words decoded by an RNN trained on instructed inner speech (and used for real-time attempted-speech decoding earlier in the session) combined with a 5-gram large-vocabulary language model. Bars represent the average number of words decoded over all trials. Error bars represent 95% CIs computed via bootstrap resampling (10,000 resamples). For both T15 and T16 the number of decoded words during verbal prompts (T15: 22.3 95% CI [12.2, 34.8]; T16: 9.3, 95% CI [7.6, 10.8]) was higher than during “clear your mind” trials (T15: 5.8, 95% CI [2.6,9.0]; T16: 4.6, 95% CI [3.6,5.4]). Additionally, in T15 the number of words decoded during autobiographical prompts (6.7, 95% CI [3.5,10.9]) was also lower than verbal prompts; this was not true in T16 (7.2, 95% CI [5.9,8.7]). **C**) Average length of trials by prompt category, showing that number of decoded words cannot be attributed to trial duration (all prompt category’s 95% CIs for average duration are overlapping for both participants). Note: progression through trials was self-paced by participants.

6 Figure S6: Nested 10-fold cross-validation window optimization procedure. Related to STAR Methods.

7 Video S1: Example real-time decoding of attempted speech and inner speech in participant T16, Related to Figure 3

8 Document S1: Tables S1,S2,S4,S5

9 Table S3: Excel file containing overview of data collection sessions, related to STAR Methods

10 Table S6: Excel file containing summary statistics, sample details, error bar descriptions, and hypothesis tests for all figures. Related to STAR Methods

11 Table S7: Excel file containing overview of supplementary training data used in online decoding evaluation sessions, related to Figure 3 and STAR Methods

## Figures and Tables

**Table:** KEY RESOURCES TABLE

REAGENT or RESOURCE	SOURCE	IDENTIFIER
**Software and Algorithms**		
MATLAB R2023b	MathWorks Inc. https://www.mathworks.com/products/matlab.html	RRID:SCR_001622
BRAND	Ali *et al* 2024	https://github.com/brandbci/brand
Python 3.9	python.org/downloads/	RRID:SCR_008394
SciPy 1.11.4	scipy.org	RRID:SCR_008058
NumPy 1.26.2	numpy.org	RRID:SCR_008633
Pandas 2.1.3	pandas.pydata.org	RRID:SCR_018214
scikit-learn 1.3.2	scikit-learn.org	RRID:SCR_002577
matplotlib 3.8.2	matplotlib.org	RRID:SCR_008624
seaborn 0.13.0	seaborn.pydata.org	RRID:SCR_018132
AWS Polly aws-cli/2.22.29	Amazon Web Services aws.amazon.com	RRID:SCR_012854
Custom analysis code	Repository provided upon acceptance.	
**Other**		
NeuroPort Neural Signal Processor	Blackrock Neurotech	https://blackrockneurotech.com/products/neuroport/
NeuroPlex E	Blackrock Neurotech	https://blackrockneurotech.com/products/neuroplex-e/
64 channel Utah Array Electrode	Blackrock Neurotech	https://blackrockneurotech.com/products/utah-array/

## Highlights

- Attempted, inner and perceived speech have a shared representation in motor cortex
- An inner speech BCI decodes general sentences with improved user experience
- Aspects of private inner speech can be decoded during cognitive tasks like counting
- High-fidelity solutions can prevent a speech BCI from decoding private inner speech
