# Supplementary figures and images for: Inner speech in motor cortex and implications for speech neuroprostheses
Source: Cell. Author manuscript; Available in PMC 2025 Aug 19. (PMC12360486; doi:10.1016/j.cell.2025.06.015)

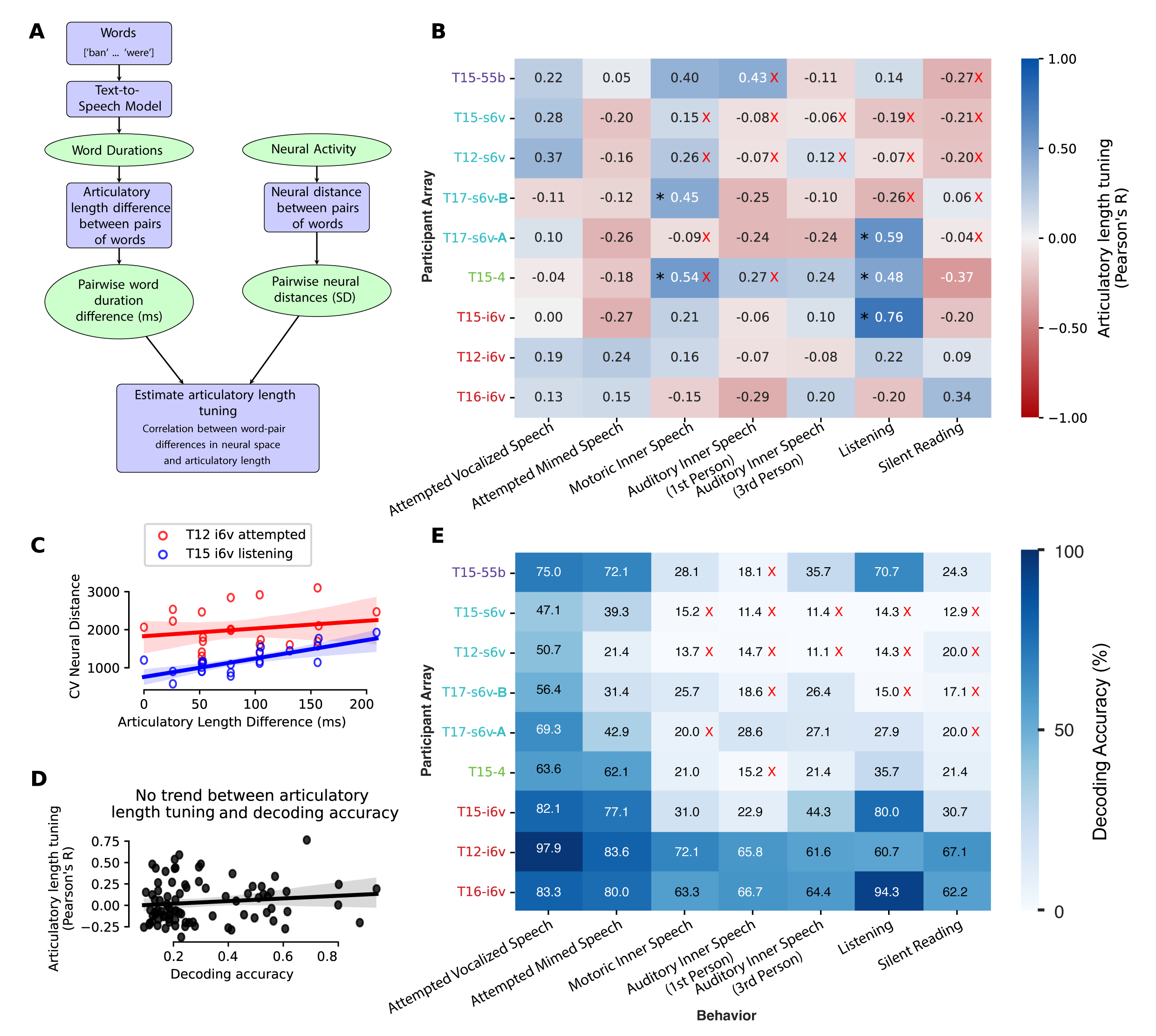

Supplement: 1 — Figure S1: Neural tuning for word duration (“articulatory length”) does not explain separability between words or the 7-word decoding performance in Figure 1, Related to Figure 1 A) Diagram of per-array analysis to estimate tuning to articulatory length. Text-to-speech models were used to generate audio for individual words. Pairwise differences between word audio durations were regressed against pairwise differences in neural distance using ordinary least squares regression. If articulatory length is encoded in neural population activity, neural differences would be explained by differences in articulatory length. B) Articulatory length tuning was computed for each array and speech behavior, matching Figure 1E. Black asterisks indicate significant tuning for articulatory length (ordinary least squares regression coefficient p-value < 0.05). Red X's indicate arrays that do not have significant decoding of seven words reported in Figure 1E. Most arrays with significant decoder performance do not significantly encode articulatory length. C) Two example scatter plots of differences for each word pair in neural distance and articulatory length shown for the highest decoding performance array and behavior (T12 i6v Attempted, red) and highest articulatory tuning array and behavior (T15 i6v Listening, blue, p-value=5.68 × 10^-5) D) Although some behaviors in some arrays do have significant tuning for articulatory length, no significant relationship between articulatory length and decoding accuracy was found across all behaviors on all arrays (p-value=0.25). E) As an additional control, the decoding analyses from Figure 1E were replicated using a shorter window of neural data matching the shortest word duration (were, 366ms). Limiting the analysis to a window aligned with the shortest word should help reduce the possibility that activity related simply to the presence vs. absence of speech contributes to word classification. Decoding results were broadly similar. This indic [file NIHMS2096625-supplement-1.tif]

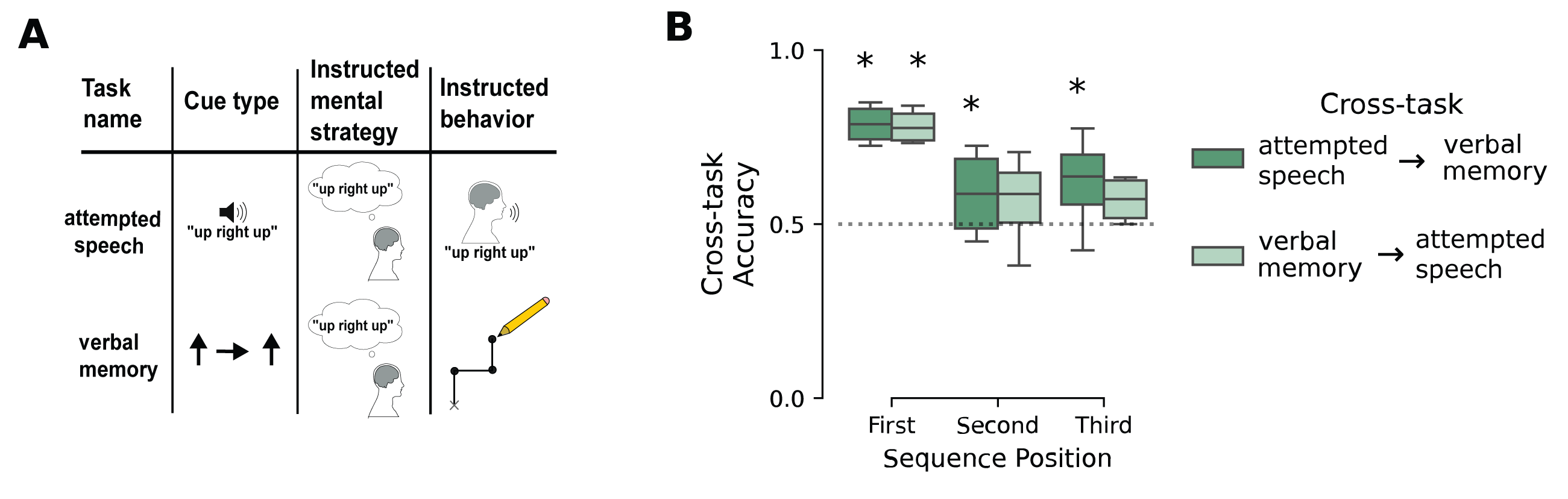

Supplement: 2 — Figure S2: Shared representation of spoken direction words and verbal memory of a motor sequence‥ Related to Figure 4 A) An attempted speaking task was compared to a motor sequence task where participant T12 was instructed to use verbal memory to remember the sequence. B) The same decoding analysis described in Figure 4B is shown for decoders trained on attempted speech and tested on verbal memory (or vice versa) to assess whether the representation of verbal short term memory and spoken direction words is shared. Box plots show cross-validated accuracy (dotted line indicates chance) and asterisks indicate above chance performance per position as assessed via a bootstrap-derived 95% CIs compared to chance level of 0.5. [file NIHMS2096625-supplement-2.tif]

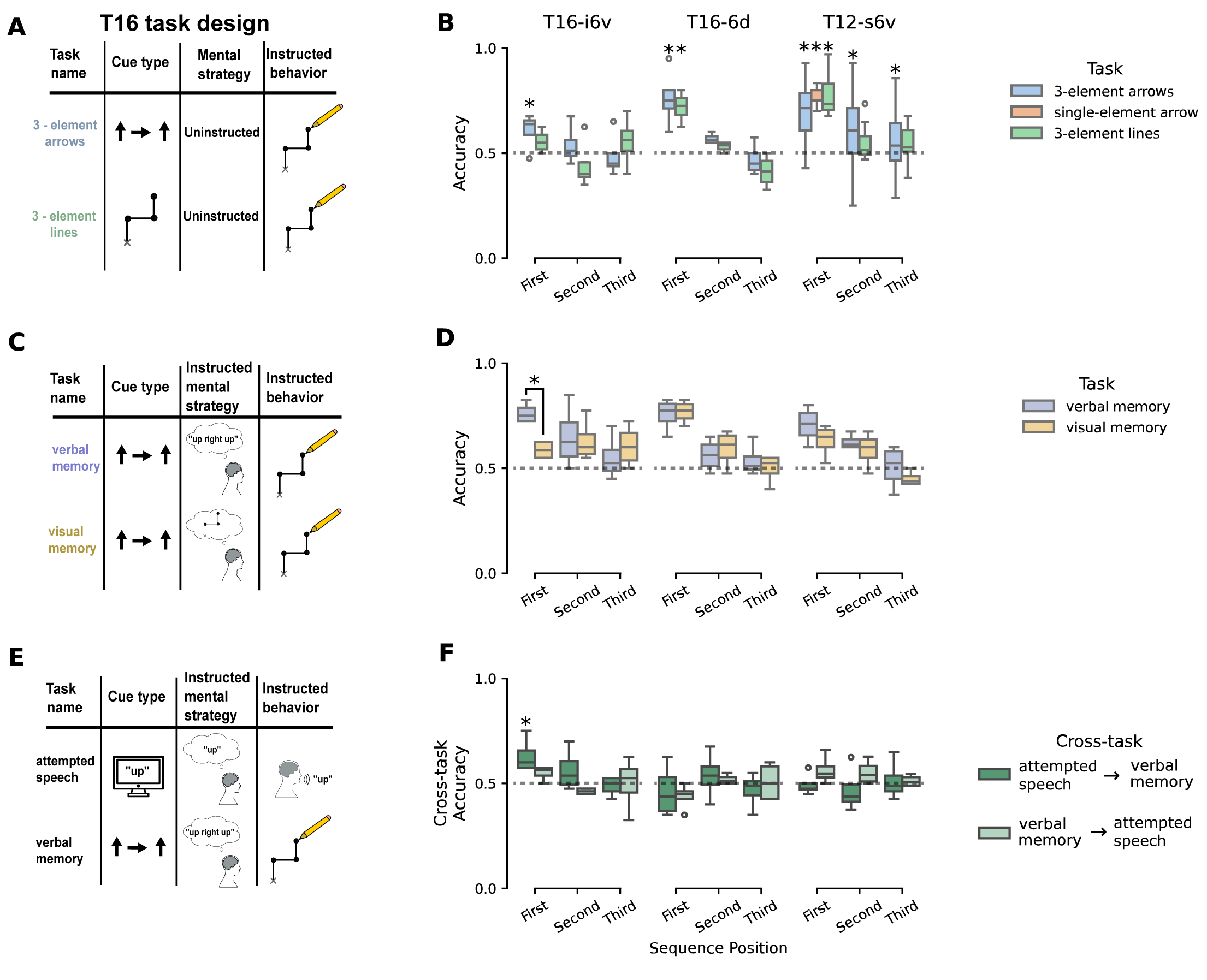

Supplement: 3 — Figure S3: Inner speech during task execution is also decodable in i6v in T16, whereas areas 6d in T16 and s6v in T12 exhibit hand-motor tuning. Related to Figure 4. A) T16 also completed the 3-element arrows and lines tasks without explicit instruction for mental strategy. Due to T16's upper-extremity paralysis, attempted drawing was instructed as the desired behavior for sequence recall (as opposed to actual drawing in participant T12). Task design for T12 is described in Figure 4A. B) Decodability of sequence position was assessed as in Figure 4. For T16, a window of neural activity from the first 1.5 seconds after the go cue was used to fit decoders. For T12 the same 2-second delay period window was used as reported in Figure 4. For area i6v in T16, only the 3-element arrow task elicited significant neural representation of the first sequence position. Our prior work75 has shown that area 6d in T16 and area s6v in T12 both encode hand-motor activity. In line with this, decoding performance was similar across all tasks, particularly for the first sequence position for T12-s6v which is distinct from T12-i6v results reported in Figure 4. Therefore, decoding results from these regions serve as a demonstration of the validity of the motor and sequencing control tasks (single-element arrow, and 3-element lines). C) T16 performed two versions of the three-element arrows task, but with explicit instruction to either use or suppress inner speech for short-term memory of the arrow sequence. D) Same as B but for tasks that only differed in instructed mental strategy. Instruction to use verbal mental strategy significantly increased decoding accuracy of the first position in area i6v in T16 (mean decoding accuracy 0.61, 95% CI 0.53–0.68) but not in areas 6d in T16 nor area s6v in T12. E) T16 was visually cued by text to speak a direction to test whether verbal short term memory in i6v had a shared representation with attempted speech. F) Same as B except decoders are traine [file NIHMS2096625-supplement-3.tif]

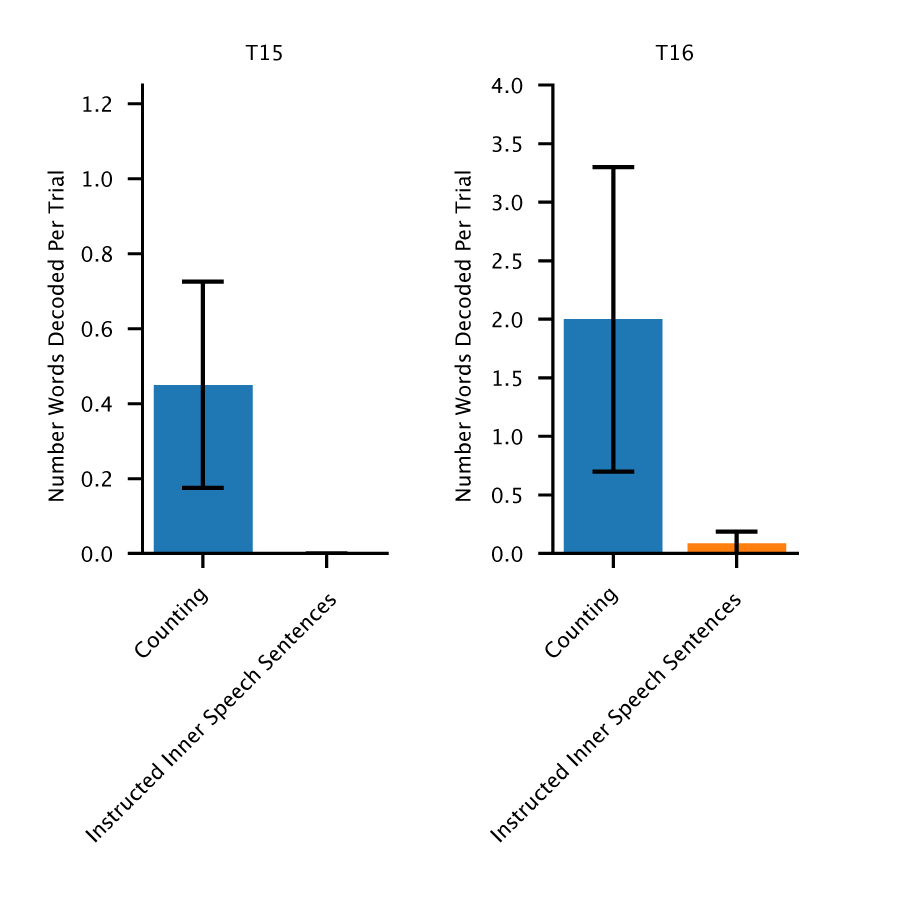

Supplement: 4 — Figure S4: Number words were more likely to be decoded during counting by a large vocabulary inner-speech BCI as compared to sentences. Related to Figure 5. Frequency of numbers decoded offline during the conjunctive-counting task (blue) compared to real-time decoded inner speech sentences (orange), which were drawn from the Switchboard corpus. For the counting task, decoding was performed offline using the same RNN and language model as in real-time decoding using a 125,000-word vocabulary (Figure 3). Unlike the analysis done in Figure 5, this involved using our standard, large-vocabulary 5-gram language model that could decode non-number words as well as numbers. As expected, numbers were decoded significantly more often from neural data recorded during the conjunctive-counting task (T15: 0.45, 95% CI [0.2, 0.75]; T16: 2.0, 95% CI [0.85,3.45]) as opposed to the instructed inner speech Switchboard sentences task (T15: 0.0, 95%CI [0.0,0.0]; T16: 0.09, 95% CI [0.0,0.2]) - with significance determined by non-overlapping confidence intervals), which further supports the conclusion that uninstructed inner speech, such as that elicited during counting, can be decoded by a speech BCI. Confidence intervals were computed via bootstrap resampling (10,000 resamplings). [file NIHMS2096625-supplement-4.tif]

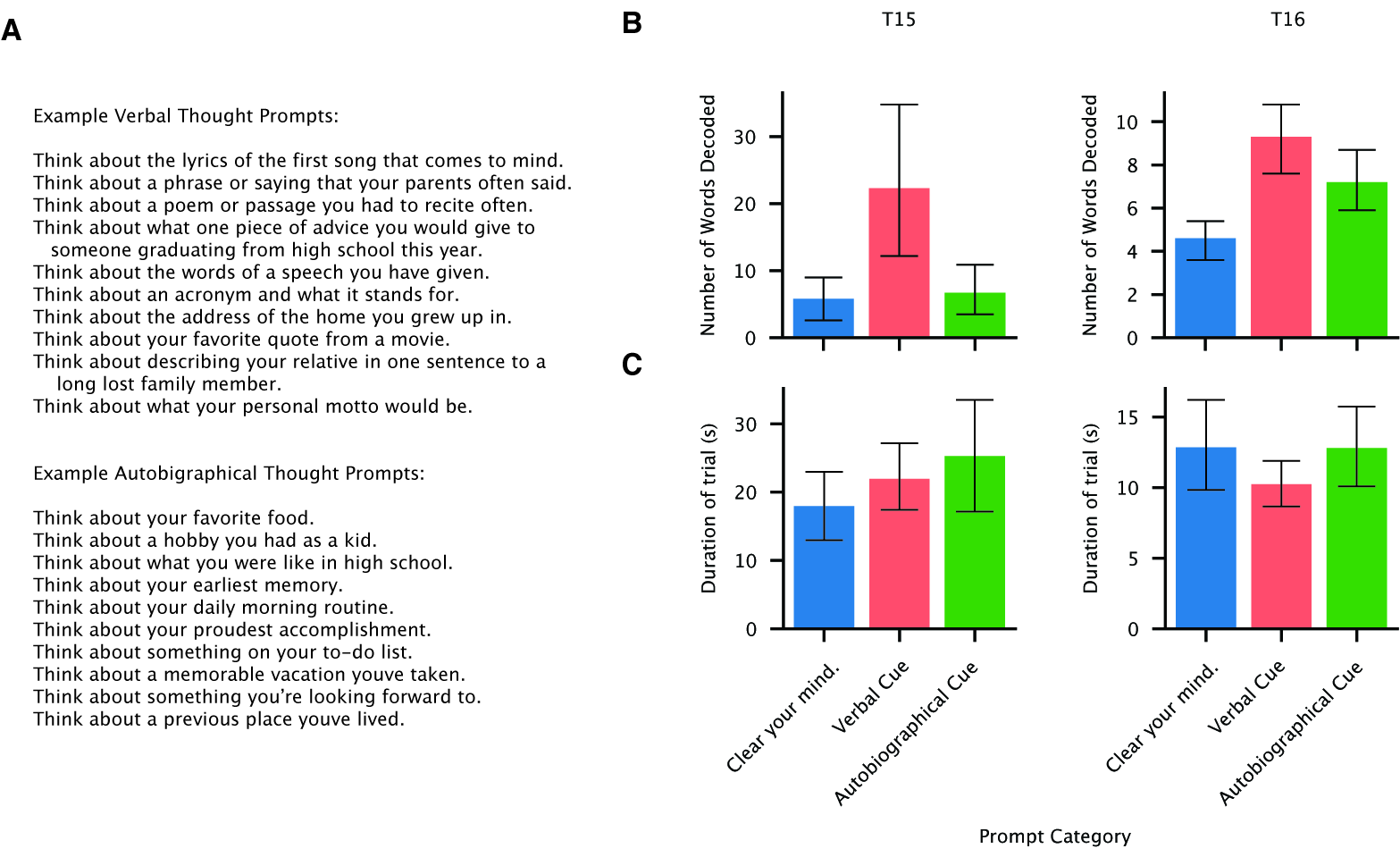

Supplement: 5 — Figure S5: Offline decoding of neural activity recorded during prompted verbal and autobiographical thought shows that more words were decoded during verbal prompts compared to “clear your mind” prompts. Related to Figure 5. A) Participants T15 and T16 engaged in a series of verbal or autobiographical thought prompts presented as text on a computer monitor. Participants were sometimes also prompted to “clear your mind”. We hypothesized that participants would engage in free-form inner speech during the verbal prompts, which would be able to be decoded by an RNN trained on instructed inner speech. Autobiographical prompts in which thought process could have taken on any different number of modalities (i.e. episodic memory or visual imagery, abstract representations, or inner speech) were also investigated. B) Number of words decoded by an RNN trained on instructed inner speech (and used for real-time attempted-speech decoding earlier in the session) combined with a 5-gram large-vocabulary language model. Bars represent the average number of words decoded over all trials. Error bars represent 95% CIs computed via bootstrap resampling (10,000 resamples). For both T15 and T16 the number of decoded words during verbal prompts (T15: 22.3 95% CI [12.2, 34.8]; T16: 9.3, 95% CI [7.6, 10.8]) was higher than during “clear your mind” trials (T15: 5.8, 95% CI [2.6,9.0]; T16: 4.6, 95% CI [3.6,5.4]). Additionally, in T15 the number of words decoded during autobiographical prompts (6.7, 95% CI [3.5,10.9]) was also lower than verbal prompts; this was not true in T16 (7.2, 95% CI [5.9,8.7]). C) Average length of trials by prompt category, showing that number of decoded words cannot be attributed to trial duration (all prompt category’s 95% CIs for average duration are overlapping for both participants). Note: progression through trials was self-paced by participants. [file NIHMS2096625-supplement-5.tif]

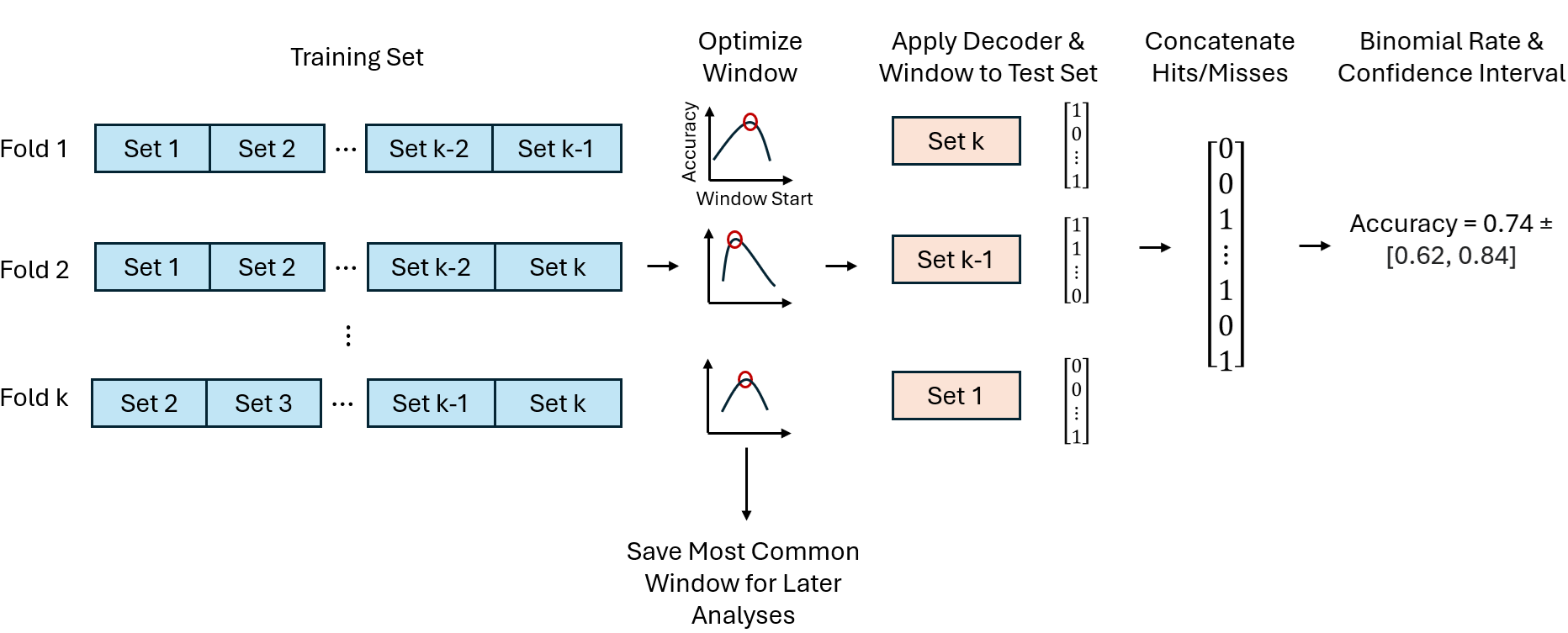

Supplement: 6 — Figure S6: Nested 10-fold cross-validation window optimization procedure. Related to STAR Methods. [file NIHMS2096625-supplement-6.tif]
