# Supplementary material for: Inner speech in motor cortex and implications for speech neuroprostheses
Source: Cell. Author manuscript; Available in PMC 2025 Aug 19. (PMC12360486; doi:10.1016/j.cell.2025.06.015)
Supplement: 7 — Video S1: Example real-time decoding of attempted speech and inner speech in participant T16, Related to Figure 3 [file NIHMS2096625-supplement-7.pdf]

Table S1: Task instructions for each verbal behavior condition.

Behavior Type		Instructions	Observed Output
Attempted speech (AS)	Attempted Vocalized Speech	"Say the word aloud (to the best of your ability)."	Able-speaking: Observed articulation and vocalization<br>Dysarthric: Some observed articulation and unintelligible vocalization<br>Anarthric: No observed articulation or vocalization
	Attempted Mimed Speech	"Mouth the word as if you were mouthing to someone across a room, without sound."	Able-speaking: Observed articulation without vocalization<br>Dysarthric: Some observed articulation without vocalization<br>Anarthric: No observed articulation or vocalization
Inner Speech (IS)	Motoric Inner Speech (1st Person Speech Motor Imagery)	"Imagine mouthing the word. Focus on what your mouth, tongue, lips, jaw and throat would be doing and how they would feel."	Able-speaking: No movement<br>Dysarthric: No movement<br>Anarthric: No movement
	1st Person Auditory Inner Speech	"Imagine uttering the word aloud. Focus on the sound you would be producing."	Able-speaking: No movement<br>Dysarthric: No movement<br>Anarthric: No movement
	3rd Person Auditory Inner Speech (Imagined Listening)	"Imagine hearing me (or someone's voice you know well) say the word, focus on the sound of my (their) voice."	Able-speaking: No movement<br>Dysarthric: No movement<br>Anarthric: No movement
Passive	Listening	"Listen to the word."	Able-speaking: No movement<br>Dysarthric: No movement<br>Anarthric: No movement
	Silent Reading	"Read the word silently without uttering or mouthing."	Able-speaking: No movement<br>Dysarthric: No movement<br>Anarthric: No movement

Table S2: Stimulus word set and their articulatory duration (estimated by text-to-speech model AWS Polly aws-cli/2.22.29, VoiceId='Joanna') and constituent phonemes, related to Figures 1, 2, 5, S1, S2, and STAR Methods.

word	duration(ms)	phonemes

ban	444	/b/ - voiced bilabial plosive<br>/æ/ - near-low front unrounded vowel<br>/n/ - voiced nasal alveolar
choice	575	/ʧ/ - voiceless postalveolar affricate<br>/ɔɪ/ - dipthong (low-mid back rounded vowel to near-high near-front unrounded vowel<br>/s/ – voiceless alveolar fricative
day	470	/d/ - voiced alveolar plosive<br>/eɪ/ – a diphthong (high-mid front unrounded vowel to near-high near-front unrounded vowel)
feel	522	/f/ – voiceless labiodental fricative<br>/iː/ high front unrounded vowel<br>/l/ – a voiced alveolar lateral sonorant
kite	522	/k/ – voiceless velar plosive<br>/aɪ̯/ - dipthong (open front unrounded vowel to near-high near-front unrounded vowel<br>/t/ – voiceless alveolar plosive
though	418	/ð̠/ - Voiced dental fricative<br>/oʊ/ - dipthong (close-mid back rounded vowel to near-high near-back rounded vowel)
were	366	/w/ - voiced labial-velar approximant<br>/ɝ/ - rhotic vowel

Table S4: Instructed delay task design for verbal behavior (Figures 1, S1) and serial-recall (Figures 4, S2, S3) par participant. Task design and trial counts reflected participant's preference and ability which varied across participant and session days. N/A indicates a task that was not run for that participant. Related to Figures 1, S1, 4, S2, S3

Task and Behavior	Delay/Intertrial Interval (ITI) Epoch duration				Go Epoch Duration				Number of repetitions				Number/type of conditions			
	T12	T15	T16	T17	T12	T15	T16	T17	T12	T15	T16	T17	T12	T15	T16	T17
Verbal Behavior: Attempted Vocalized	2s	1.5s	2s	1.5s	3s	3s	4s	3s	21	20	14	20	7 words	7 words	7 words	7 words
Verbal Behavior: Attempted Mimed	2s	1.5s	2s	1.5s	3s	3s	3s	3s	21	20	14	20	7 words	7 words	7 words	7 words
Verbal Behavior: Motoric Inner Speech	2s	1.5s	2s	1.5s	3s	3s	3s	3s	28	30	14	20	7 words	7 words	7 words	7 words
Verbal Behavior: Auditory Inner Speech (1st Person)	2s	1.5s	2s	1.5s	3s	3s	3s	3s	28	30	14	20	7 words	7 words	7 words	7 words
Verbal Behavior: Auditory Inner Speech (3rd Person)	2s	1.5s	2s	1.5s	3s	3s	3s	3s	28	30	14	20	7 words	7 words	7 words	7 words

Verbal Behavior: Passive Listening	1.5s (ITI)	0.5s (ITI)	1.5s (ITI)	0.5s (ITI)	1.5s	1.5s	1.5s	1.5s	21	20	21	20	7 words	7 words	7 words	7 words
Verbal Behavior: Silent Reading	1.5s (ITI)	0.5s (ITI)	1.5s (ITI)	0.5s (ITI)	2s	1.5s	2s	1.5s	21	20	14	20	7 words	7 words	7 words	7 words
Serial-Recall: 3 -element arrows	2.5s	N/A	2s	N/A	3s	N/A	4s	N/A	14s	N/A	20s	N/A	65 sequences	N/A	8 sequences	N/A
Serial-Recall: single-element arrows	2.5s	N/A	N/A	N/A	2.5s	N/A	N/A	N/A	30s	N/A	N/A	N/A	5 movements	N/A	N/A	N/A
Serial-Recall: 3 -element lines	2.5s	N/A	2s	N/A	4s	N/A	4s	N/A	17s	N/A	20s	N/A	17 sequences	N/A	8 sequences	N/A
Serial-Recall: verbal memory	2.5s	N/A	2s	N/A	4s	N/A	4s	N/A	20s	N/A	20s	N/A	17 sequences	N/A	8 sequences	N/A
Serial-Recall: visual memory	2.5s	N/A	2s	N/A	4s	N/A	4s	N/A	20s	N/A	20s	N/A	17 sequences	N/A	8 sequences	N/A
Serial-Recall: Attempted speech	3.5s	N/A	2s	N/A	4.5s	N/A	4s	N/A	20s	N/A	20s	N/A	9 sequences	N/A	3 sequences	N/A
Interleaved Verbal Behavior: Attempted speech	1.8s	1.5s	2s	1.5s	3s	3s	2s	3s	20	22	20	19	7 words	7 words	7 words	7 words
Interleaved Verbal Behavior: Inner Speech	1.8s	1.5s	2s	1.5s	3s	3s	2s	3s	20	44	20	38	7 words	7 words	7 words	7 words
Interleaved Verbal Behavior: Listening	1.8s	1.5s	2s	1.5s	3s	3s	2s	3s	20	22	20	19	7 words	7 words	7 words	7 words

Table S5: Resulted decoder-optimized 500ms window start time relative to go cue. N/A values indicate decoding performance was not significantly above chance. Related to figure 1,2 and STAR Methods

Participant Array	Attempted	Mimed	Motoric Inner Speech	Auditory Inner Speech	Imagined Listening	Passive Listening	Silent Reading
T15-55b	+400ms	+300ms	-200ms	N/A	+1300ms	+400ms	N/A
T15-s6v	+1100ms	+1000ms	N/A	N/A	N/A	N/A	N/A
T12-s6v	+300ms	+200ms	N/A	N/A	N/A	N/A	N/A
T17-s6v-B	+200ms	+100ms	+500ms	+100ms	+100ms	N/A	N/A

T17-s6v-A	+600ms	+1100ms	N/A	+800ms	+400ms	+500ms	+900ms
T15-4	+300ms	+300ms	N/A	N/A	+200ms	+400ms	+400ms
T15-i6v	+1000ms	+100ms	0	+100ms	+100ms	+300ms	+300ms
T12-i6v	0	0	-200ms	0	+100ms	+300ms	+700ms
T16-i6v	+2100ms	+1500ms	+1000ms	+1700ms	-400ms	+500ms	+400ms
